# Resolution of co-eluting isomers of anti-inflammatory drugs conjugated to carbonic anhydrase inhibitors from plasma in liquid chromatography by energy-resolved tandem mass spectrometry

**DOI:** 10.1080/14756366.2018.1445737

**Published:** 2018-03-14

**Authors:** Marta Menicatti, Marco Pallecchi, Silvia Bua, Daniela Vullo, Lorenzo Di Cesare Mannelli, Carla Ghelardini, Fabrizio Carta, Claudiu T. Supuran, Gianluca Bartolucci

**Affiliations:** a NEUROFARBA Department, Sezione di Scienze Farmaceutiche, University of Florence, Florence, Italy;; b Polo Scientifico, Laboratorio di Chimica Bioinorganica, University of Florence, Florence, Italy;; c NEUROFARBA Department, Sezione di Farmacologia e Tossicologia, University of Florence, Florence, Italy

**Keywords:** ERMS, linear equations of deconvolution analysis, drug plasma stability, collision breakdown curves, matrix effects

## Abstract

Rheumatoid arthritis (RA) is a chronic inflammatory disease caused by a faulty autoimmune response. Recently, it was reported that some human carbonic anhydrases (CAs) isoforms are overexpressed in inflamed synovium of RA patients. New CA inhibitors (CAIs) incorporating CA-binding moiety and the cyclooxygenase inhibitor tail (nonsteroidal anti-inflammatory drug [NSAID] type) were studied. The aim of this work is the evaluation of the chemical stability of NSAID − CAI hybrids towards spontaneous or enzymatic hydrolysis by LC-MS/MS. The analytes are isomer pairs of 6- or 7-hydroxycoumarin, their different fragment ions abundances allowed the development of a mathematical tool (LEDA) to distinguish them. LEDA reliability at ng mL^−1^ level was checked (>90%), being proved the effectiveness in the correct assignment of the isomer present in the sample. The hybrids resulted stable in all tested matrices allowing us to conclude that these compounds reach the target tissues unmodified, opening perspectives for their development in the treatment of inflammation.

## 1. Introduction

**Figure 1.**
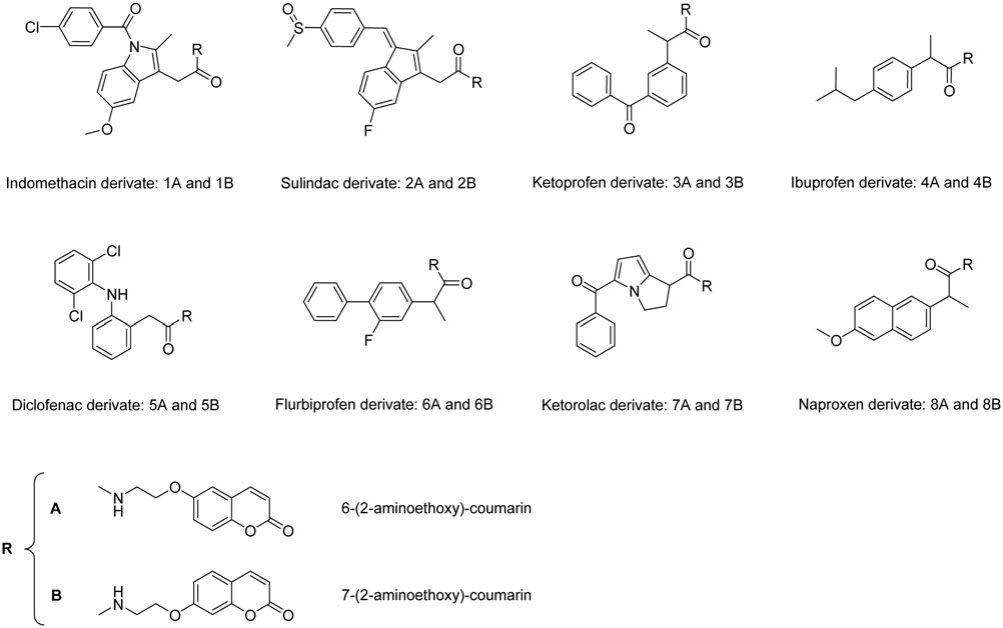
Chemical structures of NSAID − CAI hybrids.

Rheumatoid arthritis (RA) is a chronic and systemic inflammatory disease caused by a faulty autoimmune response, which primary affects the lining of the joints, thus causing erosion of the cartilage, bone damage, and joints deformity at the later stages^1^. Recently, we reported that human carbonic anhydrases (CAs, EC 4.2.1.1) isoforms IX and XII are overexpressed in inflamed synovium of patients affected by juvenile idiopathic arthritis (JIA), which is the most common RA disease in pediatric age^2^. Previous studies also showed abnormal expressions of the hCAs I, III, and IV in specimens of RA-affected patients, but only antibodies against these isoforms have been detected^3–6^. Considering thus the interplay between various hCA isoforms and the arthritis-like diseases, we developed in a recent manuscript new molecules incorporating both a CA-binding moiety (of the 6- and 7 substituted coumarin type) and a cyclooxygenase inhibitor belonging to the nonsteroidal anti-inflammatory drug (NSAID) class, which represent the most widely used pain-relief medication to date^7^. The two sections were connected by means of a physiologically cleavable linker of the amide type. This new class of low molecular weight NSAID − CA inhibitor (CAI) hybrids (Figure 1) was characterised by a selective inhibition profile *in vitro* against the hCA IX and XII^8–13^ and proved to be highly promising tools for the treatment of symptoms, such as pain, originating from RA and related diseases in an animal model of this disease. Furthermore, the inhibition of the RA over-expressed hCA isoforms IV, IX, and XII can significantly contribute to reduction of the joint local acidosis and thus to restore the humoral and cellular immunity processes which are hampered in these pathologies^14^.

The aim of this work was the evaluation of the chemical stability of such NSAID − CAI hybrids towards spontaneous or enzymatic hydrolysis in phosphate buffer solution (PBS) of human and rat plasma respectively, by application of LC-MS/MS methods. In fact, the NSAID − CAI hybrids pharmacological activity can be explained considering both the whole structure and/or their single parts (NSAID and CAI), which can be formed by hydrolysis of the intramolecular linker. Therefore, an investigation devoted to establish the chemical stability of these molecules is useful to extend the knowledge on their behavior in biological matrices and to address the research development. Furthermore, since the panel of NSAID − CAI hybrids are represented by positional isomers pairs of 6-hydroxycoumarin or 7-hydroxycoumarin, a series of LC-MS/MS methods to distinguish the isomers pairs was developed.

Analysis of isobaric molecules, and especially isomers, by tandem mass spectrometry is often complicated by the similarity between their fragmentation patterns and it is common that the same MS/MS product ions (Pi) are present in the spectra of all the isomers. Therefore, a suitable chromatographic separation between compounds should be developed in order to eliminate mutual interferences. However, the optimisation of chromatographic separation for all couples of isomers requires a lot of time and often involves the use of different instrument set up (other columns, solvents, etc.). On the other hand, several tandem mass spectrometric techniques were proposed to overcome these issues and allowing the isomers distinction^15–25^.

Unfortunately, none of these MS/MS methods were useful to solve the isomers pairs in the proposed experimental conditions. Therefore, to ensure the requested specificity of the method, a different approach, based on a series of energy resolved MS/MS experiments, was carried out. By this approach, a clear differentiation of the relative abundances of the fragment ions at different energies among all the isomers was obtained. In order to emphasise such differences, it was necessary to develop a mathematical algorithm that distinguishes the MS/MS spectra of the isomers. This algorithm (linear equations of deconvolution analysis [LEDA]) consists in the application of matrix of linear regression equations to different experimental data. In our case, the experimental data used were the abundance ratios of product vs. precursor ions selected during the MS/MS method set-up^26^^,^^27^. In this way, it was possible to resolve the MS/MS spectra assigning the correct signal to the isomer also for chromatographically unresolved peaks.

## 2. Materials and methods

### 2.1. Chemicals

Acetonitrile (Chromasolv), formic acid (MS grade), NaCl, KCl, Na_2_HPO_4_·2H_2_O, KH_2_PO_4_ (reagent grade) and verapamil hydrochloride (analytical standard, used as internal standard), ketoprofen and enalapril (analytical standard) were purchased by Sigma-Aldrich (Milan, Italy). Ketoprofen ethyl ester (KEE) was obtained by Fisher’s reaction from ketoprofen and ethanol.

MilliQ water 18 MΩ was obtained from Millipore’s Simplicity system (Milan, Italy).

PBS was prepared by adding 8.01 g L^−1^ of NaCl, 0.2 g L^−1^ of KCl, 1.78 g L^−1^ of Na_2_HPO_4_·2H_2_O, and 0.27 g L^−1^ of KH_2_PO_4_. Human plasma was collected from healthy male volunteer and the rat plasma was collected from Sprague-Dawley male rats; each plasma batch was kept at −80 °C until use.

### 2.2. Instrumental

The LC-MS/MS analysis was carried out using a Varian 1200 L triple quadrupole system (Palo Alto, CA) equipped by two Prostar 210 pumps, a Prostar 410 autosampler and an Electrospray source (ESI) operating in positive ions. Raw data were collected and processed by Varian Workstation version 6.8 software.

G-Therm 015 thermostatic oven was used to maintain the samples at 37 °C during the degradation test. ALC micro centrifugette 4214 was employed to centrifuge plasma samples.

### 2.3. LC-MS/MS methods

The chromatographic parameters employed to analyze the samples were tuned to minimise the run time. The column used was a Pursuit XRs C18 30 mm length, 2 mm internal diameter and 3 µm particle size, at constant flow of 0.25 mL min^−1^, employing a binary mobile phases elution gradient. The solvents used were 10 mM formic acid in water solution (solvent A) and 10 mM formic acid in acetonitrile (solvent B) according to the elution gradient as follows: initial at 90% solvent A, which was then decreased to 10% in 4.0 min, kept for 2.0 min, returned to initial conditions in 0.1 min and maintained for 2.0 min for reconditioning, to a total run time of 8.0 min.

The column temperature was maintained at 30 °C and the injection volume was 5 µL.

The ESI source was operated in positive ion mode, using the following setting: 5 kV needle, 42 psi nebulising gas, 600 V shield, and 20 psi drying gas at 280 °C.

The analyses were acquired in multiple reaction monitoring (MRM) using 50 ms of dwell time and the ion transitions reported in Table 1.

**Table 1.** MRM parameters.

Compound	Precursor ion (*m/z*)	Reference ion (*m/z*) [CE (V)]	Quantifier ion (*m/z*) [CE (V)]	Qualifier ion (*m/z*) [CE (V)]
**ISTD**	455	–	165 [25]	303 [25]
**1A**	545	545 [5]	312 [15]	383 [15]
**1B**	545	545 [5]	312 [15]	383 [15]
**2A**	544	544 [5]	382 [20]	313 [25]
**2B**	544	544 [5]	382 [20]	313 [25]
**3A**	442	442 [5]	280 [15]	206 [15]
**3B**	442	442 [5]	206 [15]	280 [15]
**4A**	394	394 [3]	232 [15]	206 [10]
**4B**	394	394 [3]	206 [10]	232 [15]
**5A**	483	483 [3]	206 [10]	163 [25]
**5B**	483	483 [3]	206 [10]	163 [25]
**6A**	432	432 [3]	270 [15]	206 [15]
**6B**	432	432 [3]	270 [15]	206 [15]
**7A**	443	443 [3]	210 [15]	281 [15]
**7B**	443	443 [3]	210 [15]	281 [15]
**8A**	418	418 [3]	185 [20]	256 [15]
**8B**	418	418 [3]	185 [20]	256 [15]

In order to evaluate the chemical stability of studied compounds, a dedicate LC-MS/MS method was compiled for each isomers pair with the specific transitions precursor/Pis of the ISTD and the monitored isomers.

### 2.4. Standard solutions and calibration curves

Stock solutions of analytes and verapamil hydrochloride (internal standard or ISTD) were prepared in acetonitrile at 1.0 mg mL^−1^ and stored at 4 °C. Working solutions of each analyte were freshly prepared by diluting stock solutions up to a concentration of 1.0 µg mL^−1^ and 0.1 µg mL^−1^ (working solutions 1 and 2, respectively) in mixture of mQ water:acetonitrile 50:50 (v/v). The ISTD working solution was prepared in acetonitrile at 33.3 µg mL^−1^ (ISTD solution). The spiked solutions of each analyte were prepared separately, by diluting the respective stock solutions in mQ water:acetonitrile 80:20 (v/v) solution, to obtain a final concentration of 10 µM.

A six-level calibration curve was prepared by adding proper volumes of working solution (1 or 2) of each analyte to 300 µL of ISTD solution. The obtained solutions were dried under a gentle nitrogen stream and dissolved in 1.0 mL of 10 mM of formic acid in mQ water:acetonitrile 80:20 (v/v) solution. Final concentrations of calibration levels were: 2.5, 5.0, 10.0, 25.0, 50.0, and 100.0 ng mL^−1^ of analyte in the sample.

All calibration levels were analyzed six times by LC-MS/MS system with the appropriate conditions.

### 2.5. MS/MS experiments

The Pi scan spectra were acquired in the *m/z* range from 50 to 650, scan time 600 ms; argon was used as collisional gas and the collision energy (CE) was increased stepwise in the range 5–40 V. A series of energy resolved tandem mass spectrometry (ERMS) experiments were performed to study the fragmentation of molecular species of each analyte and build its breakdown curves^26^^,^^27^. The ERMS experiments were carried out by introducing working solution 1 of each analyte, via syringe pump at 10 µL min^−1^; the protonated molecule was isolated and the abundance of Pis were monitored.

The breakdown curves were built using the relative intensities values of each signal present in the MS/MS spectra, they were obtained by averaging 50 scans for each CE.

### 2.6. Sample preparation

The sample was prepared adding 10 µL of spiked solution to 100 µL of tested matrix (PBS or human plasma or rat plasma) in microcentrifuge tubes. The obtained solutions correspond to 1 µM of analyte.

Each set of samples was incubated in triplicate at four different times, 0, 30, 60, and 120 min at 37 °C. Therefore, the matrix stability profile of each analyte was represented by a batch of 12 samples (4 incubation times ×3 replicates). After the incubation, the samples were added with 300 µL of ISTD solution and centrifuged (room temperature for 5 min at 800 *g*). The supernatants were transferred in autosampler vials and dried under a gentle stream of nitrogen.

The dried samples were dissolved in 1.0 mL of 10 mM of formic acid in mQ water:acetonitrile 80:20 solution^28^.

Three sets of six replicates for each analyte were prepared to evaluate the matrix effect (ME) and the analyte recovery^29^. The same evaluation was extended to the ISTD in order to check its reliability as quantitative reference.

The first set was prepared by mixing 10 µL of spiked solution with 300 µL of ISTD solution.

The second set was obtained by mixing 100 µL of plasma with 300 µL of acetonitrile and, after centrifugation and separation of the supernatant, by adding 10 µL of spiked solution of each analytes or 300 µL of ISTD solution.

The third set was prepared by mixing 10 µL of spiked solution with 300 µL of ISTD solution and 100 µL of plasma, then centrifuging and collecting the supernatant.

The solutions obtained were transferred in autosampler vials, dried under a gentle nitrogen stream and dissolved in 1 mL of mQ water:acetonitrile 80:20 + 10 mM of formic acid solution.

The final solutions, the three sets and the blanks, were analyzed by the LC-MS/MS methods described above.

Following the procedure described above, the expected concentrations of the samples (degradation, ME, and recovery sets) ranging about 40–50 ng mL^−1^ (depends by the MW of considered analyte), values on which the calibration curve was centred.

In order to estimate precision and accuracy of the methods, a new series of samples at three concentration levels, corresponding to 10 (low level), 25 (medium level), and 50 (high level) ng mL^−1^, were prepared for each analyte following the procedures described above from stock solutions in human plasma matrix.

### 2.7. Validation of LC-MS/MS methods

Calibration curves of analytes were obtained by plotting the peak area ratios (PAR), between analyte and ISTD quantitation ions, versus the nominal concentration of the calibration solution. A linear regression analysis was applied to obtain the best fitting function between the calibration points.

In order to obtain reliable limit of detection (LOD) and limit of quantitation (LOQ) values, the c of response and slope approach was employed^30^. The estimated SDs of responses of each analyte were obtained by the calculated SD of y-intercepts (SDY-I) of regression lines^31^.

The ME and recovery effect (RE) for each analyte were evaluated by comparing the analysis results of three sets of samples (set 1, set 2, and set 3) prepared as described in the section “Sample preparation”.

Following this procedure, the obtained results allow the determination of the ME and RE of the sample during preparation procedure by comparing the absolute peak areas of the analytes obtained from sets 1–3. The results obtained by neat standards solution in set 1 were named A, the results of standards spiked after extraction of plasma extracts were named B (set 2), and the results for standards spiked before extraction were named C (set 3), the ME and RE values can be calculated as follows^29^:

(1) $$\text{ME}(\%)=\frac{B}{A}*100$$

(2) $$\text{RE}(\%)=\frac{C}{B}*100$$

Precision and accuracy of the LC-MS/MS methods were determined at three concentration levels (low, medium, and high) for each analyte in human plasma matrix.

The precision was evaluated through the relative standard deviation (RSD%) of the quantitative data of the replicate analysis of each level. The accuracy was determined calculating the yield between the determined and added amounts.

### 2.8. LEDA algorithm

The LEDA post-processing mathematical tool was used to guarantee the identification of the isomer present in analyzed samples without their chromatographic separation. The algorithm is based on the consideration that each MS/MS spectrum might be represented as the sum of contribution of each isomer present in the sample. In order to obtain reliable data, the relative abundances of the different Pis were calculated with respect to the reference ion (Ri) abundance, so that possible misleading results due to compound-dependent different Pi yields are avoided. For this purpose, the available signal of the precursor ion was acquired as Ri which allowed us to obtain the characteristic ratios among the selected Pis for isomer speciation. The CE value of the Ri signal, leading to its highest intensity, without the occurrence of any fragmentation process for all the isomers, was reported in Table 2. By this approach, the ratio between the abundance of each Pi acquired and the abundance of Ri represents the yield of the Pi formation at the considered CE.

**Table 2.** The characteristic abundance ratios of Pi vs. Ri selected in the MRM methods and their relative standard deviations (RSD).

Isomers	383 *m/z*/Ri	RSD%	312 *m/z*/Ri	RSD%
1A	0.06	9	0.95	5
1B	0.01	18	1.07	10
	382 *m/z*/Ri		313 *m/z*/Ri	
2A	0.40	10	0.15	8
2B	0.17	5	0.20	11
	280 *m/z*/Ri		206 *m/z*/Ri	
3A	1.17	3	0.23	10
3B	0.37	3	0.69	2
	232 *m/z*/Ri		206 *m/z*/Ri	
4A	1.13	2	0.44	2
4B	0.19	9	0.92	1
	206 *m/z*/Ri		163 *m/z*/Ri	
5A	1.11	3	0.30	1
5B	1.24	3	0.11	6
	270 *m/z*/Ri		206 *m/z*/Ri	
6A	2.31	12	0.40	13
6B	1.26	7	1.54	5
	281 *m/z*/Ri		210 *m/z*/Ri	
7A	0.27	5	0.67	2
7B	0.05	18	0.66	2
	256 *m/z*/Ri		185 *m/z*/Ri	
8A	2.47	3	2.41	6
8B	0.41	12	5.03	3

Therefore, knowing the characteristic abundance ratios of pure isomer, a deconvolution of these spectra is possible based on a series of linear regression equations as follows:

(3) $${\left(\frac{\text{Pi}}{\text{Ri}}\right)}_{m}=\sum_{x=1}^{n}{\left(\frac{\text{Pi}}{\text{Ri}}\right)}_{x}*{\left[\%\right]}_{x}$$

where:

(Pi/Ri)*_m_* is the abundance ratio between the product ion (Pi) vs. reference ion (Ri) measured (m) in the sample.

(Pi/Ri)*_x_* is the characteristic abundance ratios between the Pi vs. Ri of pure isomers.

[%]*_x_* is the concentration (%) of isomers in the sample.

In order to resolve the mixtures of ***n*** isomers, a matrix, composed of ***n*** linear regression (Equation 3) obtained from ***n*** different experimental data, is required. In our case, the experimental data to be used were the abundance ratios of Pi/Ri selected during MS/MS method set-up. Therefore, at least two equations are required to compose the LEDA to determine the relative proportions of individual isomers in the standard mixtures. The deconvolution was performed by applying the algorithm to the area abundances obtained from the integrated peak intensities of each MRM channel.

The characteristic abundance ratios were obtained analysing the working solution 2 of each pure isomer by LC-MS/MS methods describe above. The ratios between Pi vs. Ri selected in the MRM methods were calculated and the resulted values were reported in Table 2.

Since the LEDA algorithm was proposed to ensure the specificity of the LC-MS/MS method, its performances were checked by processing the MS/MS data obtained from the analysis of the calibration levels and verified by its application in post-processing analysis of the samples.

## 3. Results and discussion

### 3.1. Chromatographic separation

**Figure 2.**
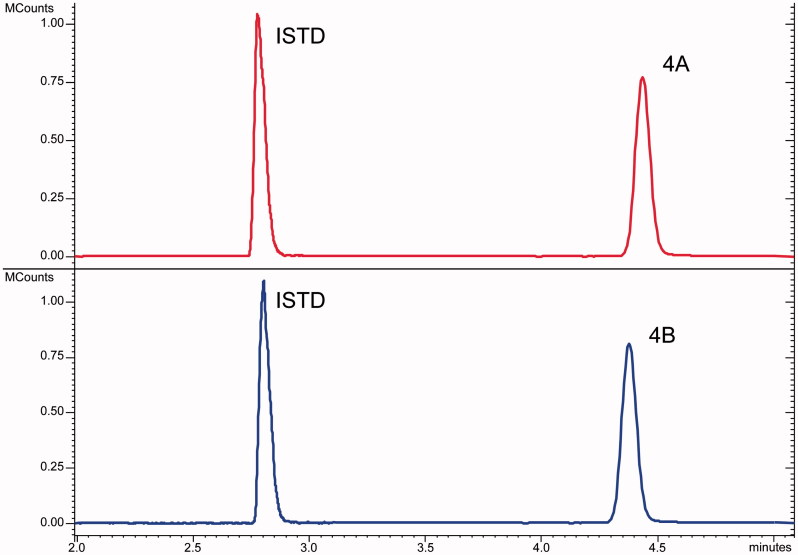
Chromatographic profiles of LC-MS/MS analysis of 4A (top) and 4B (bottom) isomers.

The chromatographic profiles obtained by the analysis of the samples with proposed LC-MS/MS methods show a good resolution between the ISTD and the analytes but did not allow the separation among the isomers peaks. A typical comparison of LC-MS/MS analysis of an isomers pair was reported in Figure 2. The peak parameters (i.e. retention times, peaks width, efficiency, etc.) for each analyte were calculated and reported in Supplemental material (Table ST1).

The evaluation of the chromatographic data confirms that the used LC conditions did not able to resolve any couples of isomers. Therefore, in order to properly identify the CAI hybrid species processed in the sample and to ensure its proper monitoring during the degradation experiment, the MS/MS features for each analyte were explored.

### 3.2. Collision-induced dissociation study

The isomeric compounds under study (see Figure 1) in ESI-MS conditions show the production of abundant protonated molecules, detected at the same *m/z* value for each isomers pair, and only a few fragment ions at very low relative abundance (less than 2%). Consequently, to obtain a signal characterisation of the analytes, some MS/MS experiments were needed. In order to evidence possible different intrinsic molecular stability of the analytes, the study of fragment ion yields present at different CEs (breakdown curves) was performed. To achieve valid and well reproducible fragmentation pattern, the collision gas pressure was critically evaluated, based on the results obtained and discussed in a previous paper^26^.

The fragmentation pathway of the studied compounds generally showed the bond cleavages both at the oxy-coumarin and at the amide group linked to the coumarin moiety. Obviously, other fragments were detected but without any diagnostic value for isomers characterisation. Usually, these Pis were originated from the NSAID-fragment of the molecule, which it is common in the isomers pair.

**Figure 3.**
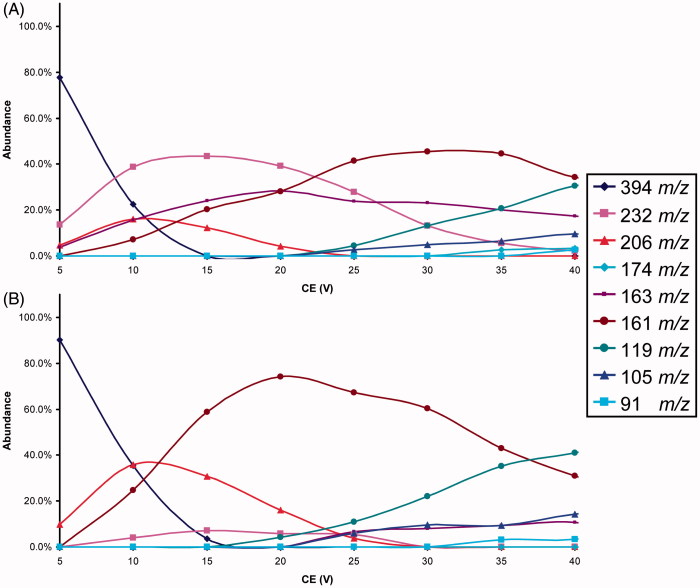
Breakdown curves of 4A (top) and 4B (bottom) isomers.

The typical graphic comparison of the breakdown curves obtained from the ERMS experiments processed data of an isomer pair was reported in Figure 3. The complete series of the breakdown curves for each isomer pairs were reported in Supplemental material (SF1–SF8). The analysis of these data it allows the selection, for each couple of isomers, of the most characteristic Pis and their CE to arrange the respective MRM methods.

### 3.3. Linearity and LOD

The obtained linear regressions coefficients, the R-squared (*R*^2^) and the estimated LOD values for each analyte are reported in Table 3.

**Table 3.** Linear regressions data, *R*^2^, and LOD values obtained for each analyte.

Compound	Slope (PAR/ng mL^−1^)	Intercept (PAR)	*R*^2^	LOD SDY-I (ng mL^−1^)
**1A**	0.012	−0.004	0.999	1.7
**1B**	0.009	0.001	0.999	0.6
**2A**	0.012	0.016	0.999	2.2
**2B**	0.005	0.010	0.997	3.7
**3A**	0.031	0.010	0.999	0.7
**3B**	0.021	0.014	0.999	0.8
**4A**	0.071	0.047	0.999	0.9
**4B**	0.045	0.011	0.999	1.0
**5A**	0.018	0.020	0.999	1.9
**5B**	0.024	0.015	0.999	1.7
**6A**	0.025	0.017	0.999	2.4
**6B**	0.013	0.005	0.999	1.4
**7A**	0.007	0.006	0.999	1.1
**7B**	0.004	0.006	0.998	2.9
**8A**	0.017	0.016	0.999	1.0
**8B**	0.029	0.018	0.999	0.8

It is worth to emphasise how the obtained slope values for each isomers pair are significantly different; therefore, it is crucial to have a correct assignment of the signal to the processed isomer, in order to avoid quantitative errors.

Despite the noted slope differences between the couples of isomers, the corresponded LOD values were comparable, confirming that the detectability depends of the stability of the signal not on its absolute intensity. In fact, following the SD of response and slope approach, the LOD values were calculated on the SD of the signal, neglecting the intensity values or evaluation of the background noise that can be variable and dependent upon several factors. In this way, it was possible to obtain LOD values each time that the calibration curve was performed, enabling the monitoring of the instrumental performances between different analyses batches. Moreover, the obtained LOD values strengthened the reliability of the low concentration levels chosen for the calibration curves.

### 3.4. Matrix effects, recovery, and LEDA performances

The values of ME and RE were calculated as reported in the section ‘Validation of LC-MS/MS methods’ and shown in Table 4. The obtained RE values (yield major than 75%) demonstrated that the analytes were correctly extracted from the plasma matrices and consequently the LC-MS/MS methods employed were suitable for their determination in samples. Also the calculated ME values showed that the biological matrices did not affect the mechanism of ionisation of the studied analytes. The obtained ME and RE values for the ISTD resulted above the 95% for both the tested matrices, confirming that its use is appropriate for monitoring the studied analytes.

**Table 4.** Data results of ME and RE, obtained in human and rat plasma samples, and evaluation of LEDA performances.

	Human plasma		Rat plasma			
Compound	ME%	RE%	ME%	RE%	LEDA Purity%	SD%
**ISTD**	98	95	96	100	n.d.	n.d.
**1A**	93	94	74	88	100	4.0
**1B**	84	97	71	88	91	1.2
**2A**	84	91	78	96	96	2.8
**2B**	95	96	98	98	94	2.5
**3A**	97	97	97	93	100	1.0
**3B**	94	94	94	98	100	1.1
**4A**	78	86	93	81	100	0.1
**4B**	67	95	82	98	100	0.7
**5A**	87	94	72	85	99	1.3
**5B**	94	95	87	95	100	1.0
**6A**	97	92	100	92	96	0.2
**6B**	98	78	97	75	100	3.5
**7A**	97	96	98	97	100	1.7
**7B**	96	96	98	99	92	0.9
**8A**	100	95	99	100	100	0.8
**8B**	95	89	95	87	100	0.9

n.d.: not determined.

**Figure 4.**
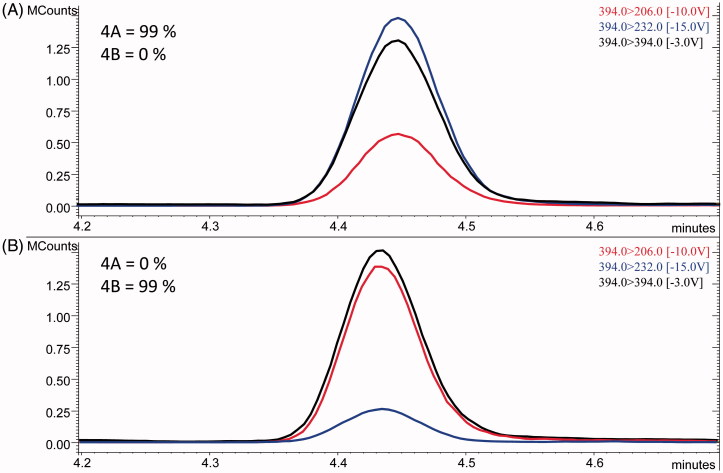
Example of chromatographic peaks obtained by LC-MS/MS analysis of calibration level solutions of compounds **4A** (top) and **4B** (bottom). LEDA purity assignment of each profile was reported (top left).

The LEDA performances were evaluated processing the MS/MS data, obtained from the calibration levels of each analyte, identifying which isomer was present, as well as its purity. In fact, under described instrumental conditions, the chromatographic peaks of each isomers pair were unresolved. Hence, the algorithm had to elaborate the corresponding isomer peak area of each MRM channels, separating their components and assigning the correct abundance to the identified isomers (see Figure 4). The purity values were calculated by the application of LEDA and represent the percentage of the MS/MS signal assigned to the processed pure isomer, with a threshold that can be represented by the SD. The obtained purity data and corresponded SD are reported in Table 4. The LEDA purity results (>90%) demonstrate that the post-processing algorithm was able to distinguish between the isomers and ensure the correct signal assignment. In order to verify its reliability, all samples involved in the stability test were processed with the LEDA tool. During the processing, in some cases, we observed that LEDA results gave a low purity of the isomer identified. However, the quantitative data, obtained by the abundances ratios between quantitation ion and ISTD, were in accordance with the expected ones. Carefully examining those MRM signals, it was noted that these occurrences can be determined by Ri abnormal signals. The abundance of Ri signal could be affected by possible matrix interferences that may compromise the absolute value of the Pi/Ri ratios. However, this effect was distributed to all the Pi ratios employed to compile the LEDA algorithm; hence, the isomer assignment was not altered. Probably, the unassigned signal corresponds to the abundance of precursor ion coming from the matrix interferences.

**Figure 5.**
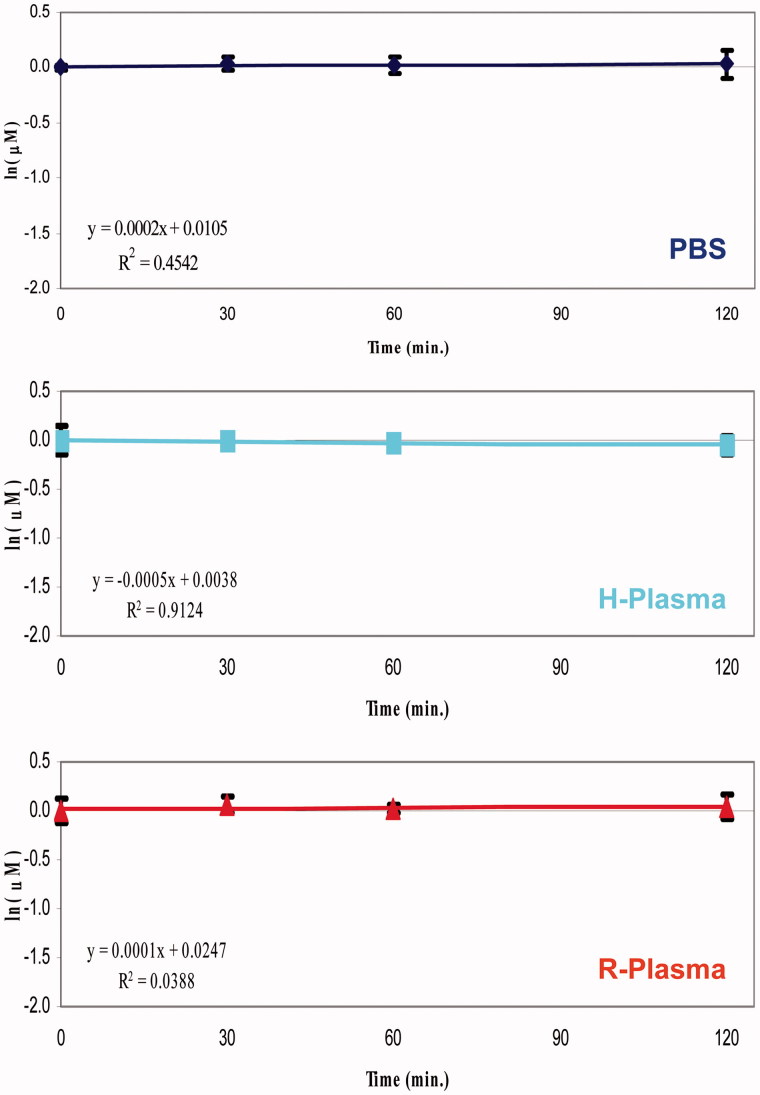
Degradation plots of 4B isomer in phosphate buffer solution (PBS), human plasma (H-Plasma) and rat plasma (R-Plasma) matrices.

### 3.5. Accuracy and precision of LC-MS/MS method

The accuracy and precision results obtained analysing the series of human plasma samples, at three concentration levels, for each studied compound using the LC-MS/MS methods described above are reported in Table 5.

**Table 5.** Results of accuracy and precision of the LC-MS/MS methods.

	Low level		Medium level		High level	
Isomer	Accuracy (%yield)	Precision (RSD%)	Accuracy (%yield)	Precision (RSD%)	Accuracy (%yield)	Precision (RSD%)
1A	99.6	5.8	96.6	1.1	97.7	4.3
1B	99.3	3.7	99.6	3.0	98.9	2.4
2A	97.4	1.1	93.3	4.8	96.5	6.0
2B	98.1	10.9	87.7	9.4	94.6	1.9
3A	99.7	4.2	96.7	4.9	99.8	3.6
3B	95.9	7.5	98.0	7.5	99.7	3.1
4A	99.7	0.7	96.8	1.5	98.8	2.6
4B	93.6	1.5	96.1	3.8	99.5	3.7
5A	99.5	2.8	95.4	6.1	96.5	2.0
5B	91.2	3.8	98.4	7.1	96.3	2.6
6A	93.8	1.0	98.8	7.4	94.2	3.8
6B	91.6	6.4	99.6	2.7	97.0	5.0
7A	99.5	15.8	98.2	5.2	98.2	6.9
7B	99.8	12.8	97.3	5.1	93.6	6.9
8A	92.1	3.9	98.0	5.3	99.7	5.6
8B	99.1	6.6	99.0	4.5	98.7	1.6

These evaluations were carried out with human plasma since it was easily available and it did not show significant differences on the ME and RE values compared to rat plasma.

The obtained accuracy (between 87.7% and 99.8%) and precision (RSD lower than 10%) data for all the analytes allows to be confident on their determination in the degradation experiment samples. Just a couple of compounds show the RSD major than 10%, but these values occurred at lower level tested only.

### 3.6. Chemical stability tests

An investigation on the chemical stability of compounds under study in PBS, human and rat plasma was performed for evaluating their susceptibility to spontaneous or enzymatic hydrolysis.

In order to establish the hydrolytic activity of the employed plasma batches, two reference compounds were tested, following the procedure reported in the section ‘Validation of LC-MS/MS methods’: KEE (half-life <2 h) and enalapril (half-life <0.5 h) to verify the human and rat plasma activity, respectively^32^^,^^33^.

The stability profiles were obtained by monitoring the variation of analyte concentration at different incubation times in PBS or human or rat plasma samples. Generally, when the substrate concentration was smaller than the Michaelis–Menten constant (K_M_), the enzymatic degradation rate is described to a first-order kinetics^34^. Therefore, plotting the natural logarithm of the quantitative data versus the incubation time, a linear function can be used and its slope represents the degradation rate constant (*k*). The degradation plots of KEE and Enalapril showed an important decay rate, and their calculated half-life (*t*_1/2_ values 95 min. and 22 min respectively), comparable to what reported in the literature^32^^,^^33^, demonstrate that the employed human and rat plasma batches were enzymatically active (Supplemental material SF9–SF10). Conversely, the studied compounds did not exhibit significant degradation in all matrices tested so far.

All degradation plots of references and studied compounds were reported in the Supplemental material (SF11–SF26). The half-life of each tested compound was calculated as follows:

(4) $$t_{1/2}=\frac{\ln(0.50µM)}{k}$$

The obtained *k* values of the studied compounds were close to 0, consequently extremely high *t*_1/2_ values were calculated (Figure 5). Under the proposed experimental conditions, a half-life of the 240 min is considered to be negligible, therefore, it is reasonable to consider that the obtained values of the half-life for all NSAID − CAI hybrids were >240 min. The *t*_1/2_ values of references and studied isomers are reported in the Supplemental material (Table ST2).

## 4. Conclusions

Considering the interest of the new NSAID − CAI hybrids, the development of analytical procedures able to evaluate their chemical stability *in vitro* conditions is an essential tool for the estimation of their bioavailability. As a matter of fact, LC-MS is the analytical system of choice in this field, but the complexity of natural substrate (cell media, plasma, etc.) can lead to misleading results for co-elution with interfering compounds. In the present study, a MS/MS procedure was developed and some important parameters affecting the analytical output were investigated. The optimal experimental conditions, in terms of CE and specific Pis, were found and employed to perform the LC-MS/MS experiments. On the other hand, the NSAID − CAI hybrids were grouped in couples of positional isomers incorporating 6-hydroxycoumarin or 7-hydroxycoumarin. Therefore, a mathematical algorithm (LEDA) was applied to separate the MS/MS signals, from unresolved chromatographic peaks, and to assign the correct abundance of the isomer present in the sample at ng mL^−1^ level. Its reliability was checked, being proved the effectiveness that allows the correct assignment of the processed isomer present in the sample, and ensuring its proper monitoring. In fact, the obtained data from the calibration curves showed different slope values for each isomers pair; demonstrating that it is important to have a correct assignment of the signal of the processed isomer, in order to avoid quantitative errors. Although positional isomers can in principle be separated by ion mobility (IM), prior to mass spectrometry analysis for quantitation in online LC-IM-MS/MS measurements^35^^,^^36^, this approach has the advantage that isomers can be quantified without the need of additional specialised IM instrumentation. Moreover, because the isomers do not need to be resolved by LC, this approach has the other advantage that faster LC separation conditions can be used to quantify the analytes than are typically required.

The obtained results demonstrated that the developed LC-MS/MS methods were suitable for the determination of studied compounds at ng mL^−1^ level in PBS or human or rat plasma samples and for describing their degradation profiles. The calculated data of precision, accuracy, and LOD values for all the analytes are suitable to have confidence on their degradation results. Furthermore, the results on the evaluation of MEs and REs showed that the procedure of preparation of samples was reliable (RE larger than 75%) and did not affect the ionisation efficiency (ME larger than 70%). The NSAID − CAI hybrids were stable in all the tested matrices at the experimental conditions used in this work. Therefore, the collected information indicate that these compounds reach the target tissues unmodified, opening new perspective on the development of NSAID − CAI hybrids with potential applications in the treatment of inflammation.

## Supplementary Material

IENZ_1445737_Supplementary_Material.pdf
