# Supplementary material for: Resolution of co-eluting isomers of anti-inflammatory drugs conjugated to carbonic anhydrase inhibitors from plasma in liquid chromatography by energy-resolved tandem mass spectrometry
Source: J Enzyme Inhib Med Chem. 2018 Mar 14;33(1):671–9. doi: 10.1080/14756366.2018.1445737 (PMC6010112; doi:10.1080/14756366.2018.1445737)
Supplement: IENZ_1445737_Supplementary_Material.pdf [file IENZ_A_1445737_SM6997.pdf]

Supporting Information

Resolution of co-eluting isomers of anti-inflammatory drugs conjugated to carbonic anhydrase inhibitors from plasma in liquid chromatography by energy-resolved tandem mass spectrometry

Marta Menicatti[1], Marco Pallecchi[1], Silvia Bua[1], Daniela Vullo[3], Lorenzo Di Cesare Mannelli[2], Carla Ghelardini[2], Fabrizio Carta[1], Claudiu T. Supuran[1], and Gianluca Bartolucci[1].

1) NEUROFARBA, Pharmaceutical Sciences section, University of Florence, Via Ugo Schiff 6, 50019 Sesto Fiorentino, Florence, Italy

2) NEUROFARBA, Pharmacology and Toxicology section, University of Florence, V.le G. Pieraccini 6 , 50139 Florence, Italy

3) Polo Scientifico, Laboratorio di Chimica Bioinorganica, University of Florence, Rm. 188, Via della Lastruccia 3, 50019 Sesto Fiorentino, Florence, Italy

ST1: Chromatographic peak parameters obtained for ISTD and the studied analytes using the conditions reported in 2.5 section.

	**Rt (min)**	**± 2 SD (min)**	**Width (min)**	**N (plates)**	**k**	**α**	**R**
**ISTD**	2.79	0.03	0.05	16435	3.99		
**1A**	4.31	0.03	0.06	31100	6.69	1.01	0.4
**1B**	4.26	0.02	0.06	31341	6.61		
**2A**	3.53	0.02	0.05	24750	5.30	1.01	0.2
**2B**	3.51	0.02	0.05	25257	5.26		
**3A**	3.81	0.03	0.06	22928	5.80	1.01	0.3
**3B**	3.77	0.02	0.06	23360	5.73		
**4A**	4.44	0.01	0.06	31436	6.92	1.01	0.4
**4B**	4.39	0.01	0.06	32273	6.84		
**5A**	4.62	0.03	0.08	17767	7.26	1.01	0.3
**5B**	4.58	0.02	0.08	20099	7.19		
**6A**	4.15	0.02	0.06	24850	6.41	1.01	0.3
**6B**	4.10	0.02	0.07	21538	6.33		
**7A**	3.63	0.02	0.05	25394	5.48	1.01	0.3
**7B**	3.60	0.01	0.06	22543	5.42		
**8A**	3.79	0.02	0.05	30078	5.76	1.01	0.3
**8B**	3.76	0.03	0.05	26624	5.71		

Rt: Retention time

SD: Standard Deviation

Width: Peak width

N: Efficiency

k: Retention factor

a: Selectivity

R: Resolution

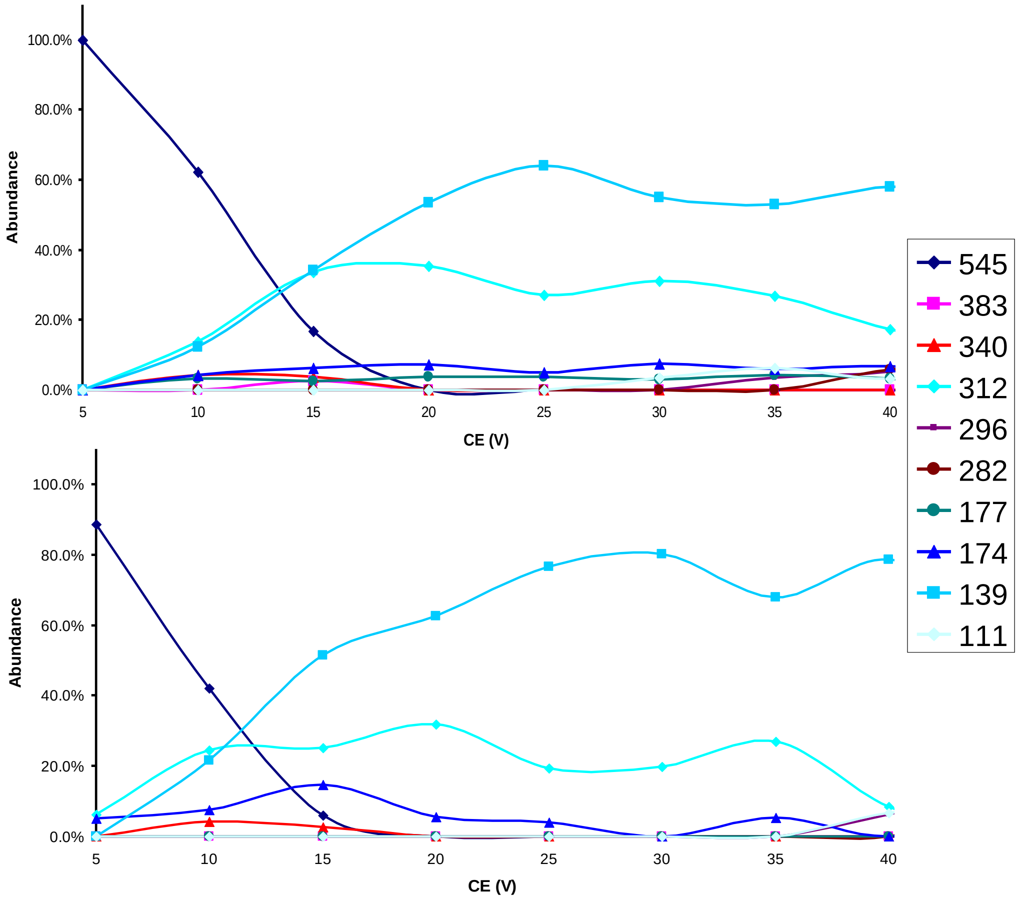

SF1: Breakdown curves for 1a-1b isomer pairs.

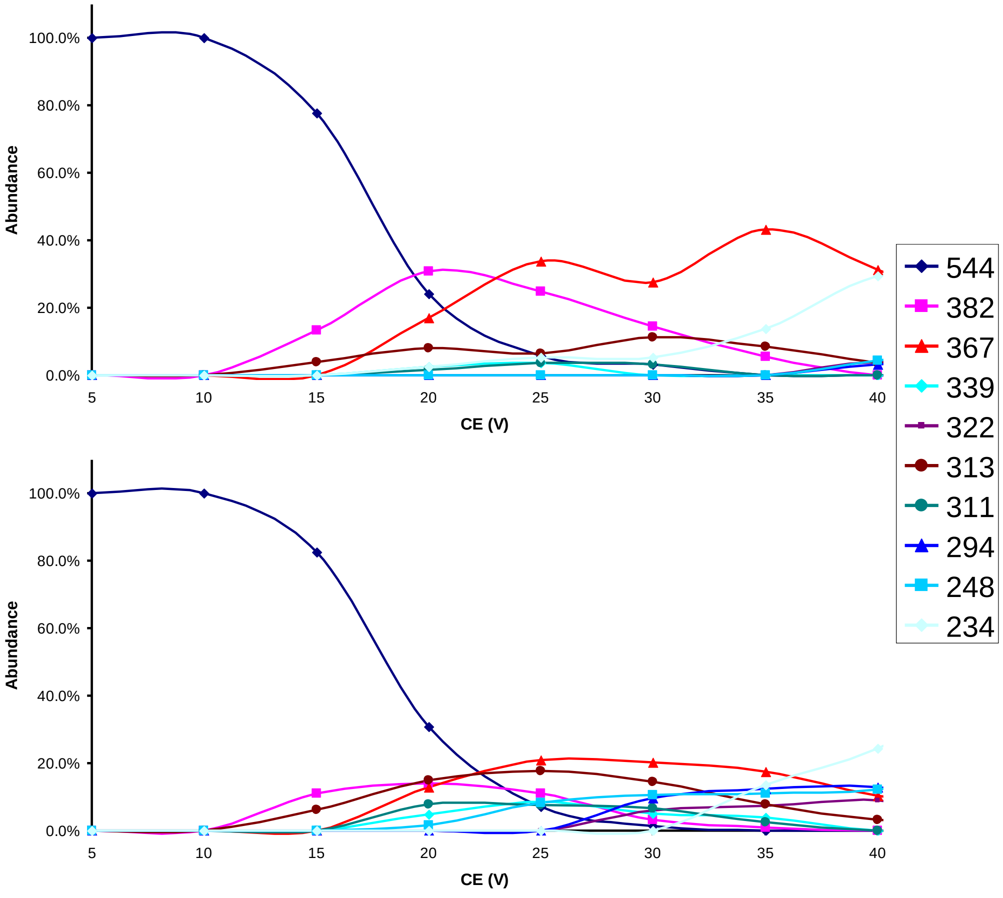

SF2: Breakdown curves for 2a-2b isomer pairs.

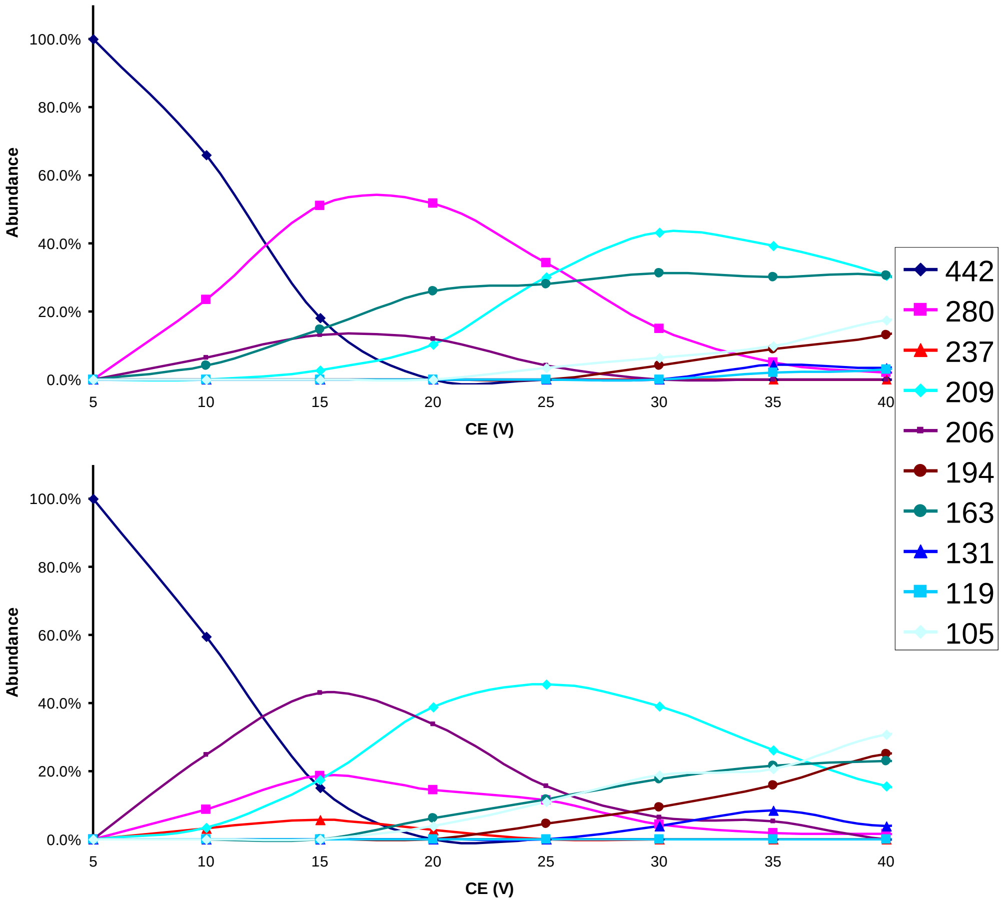

SF3: Breakdown curves for 3a-3b isomer pairs.

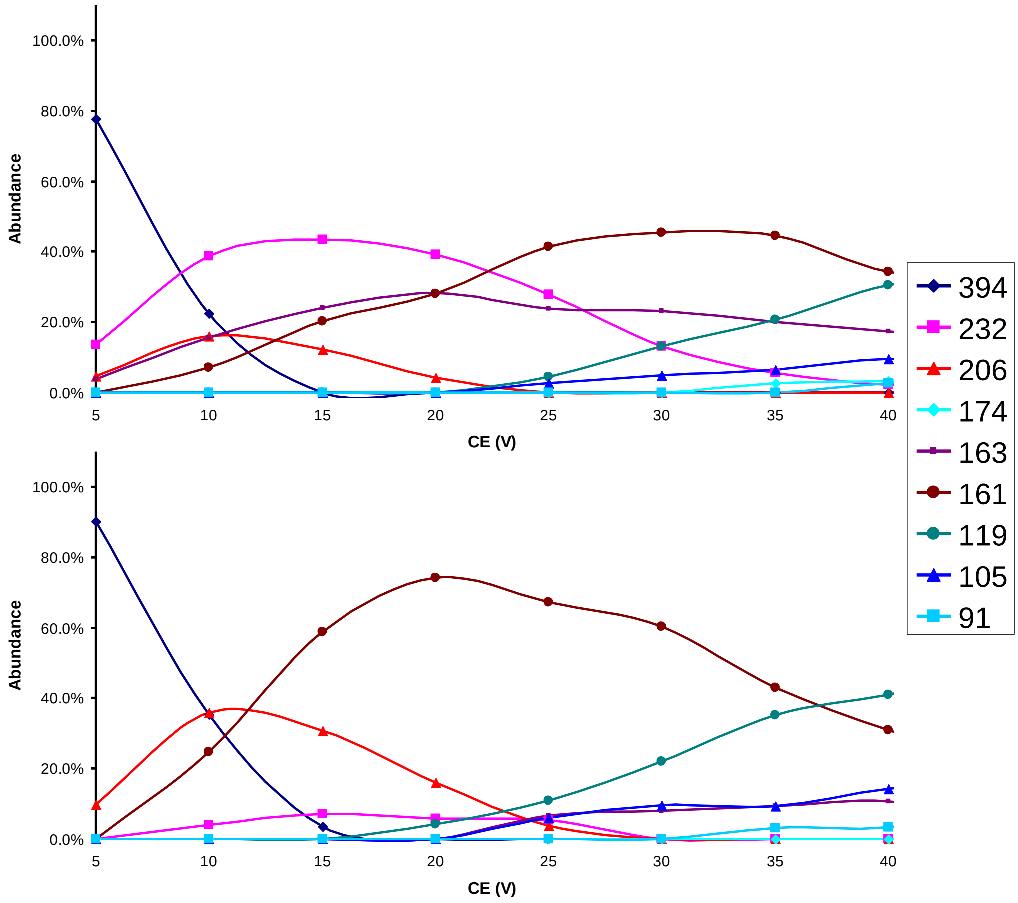

SF4: Breakdown curves for 4a-4b isomer pairs.

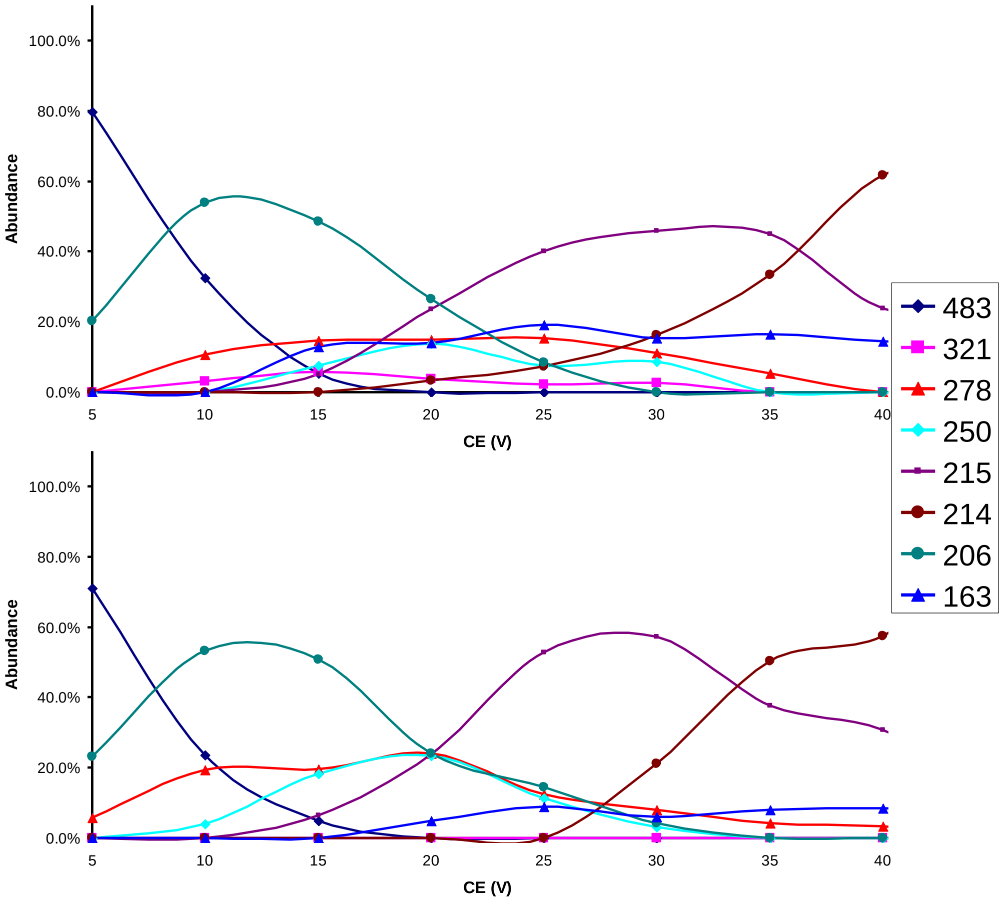

SF5: Breakdown curves for 5a-5b isomer pairs.

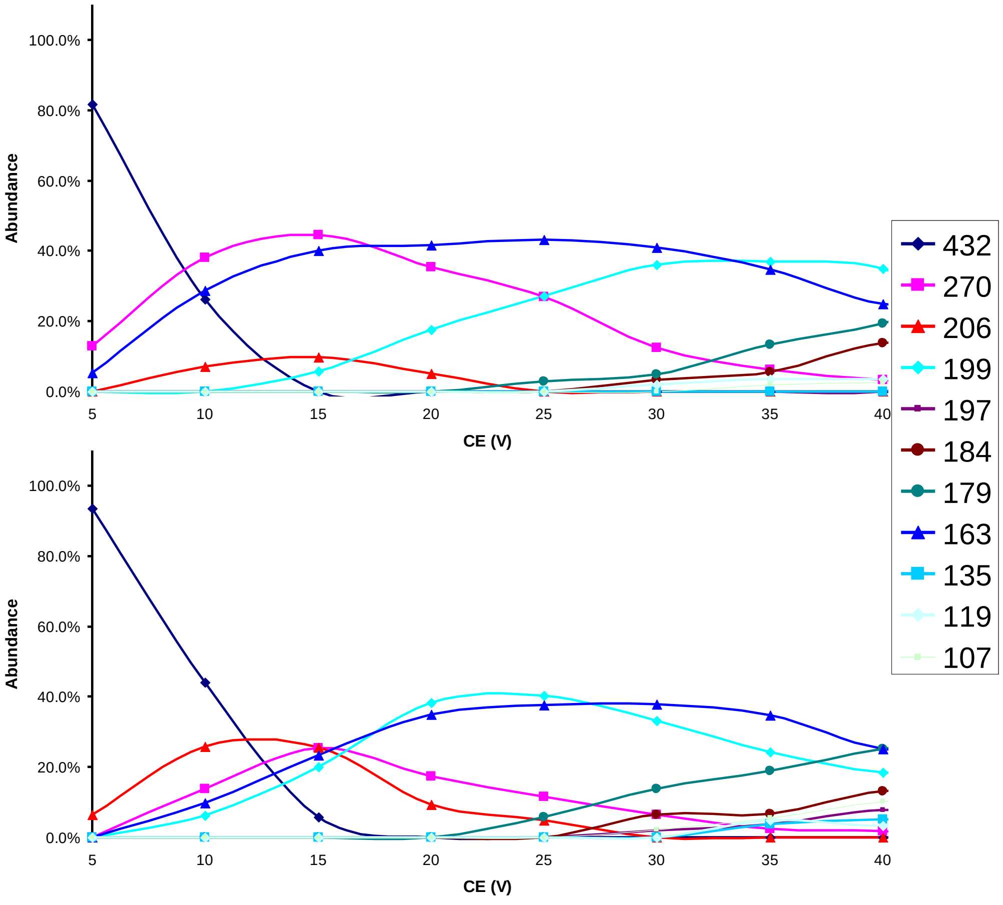

SF6: Breakdown curves for 6a-6b isomer pairs.

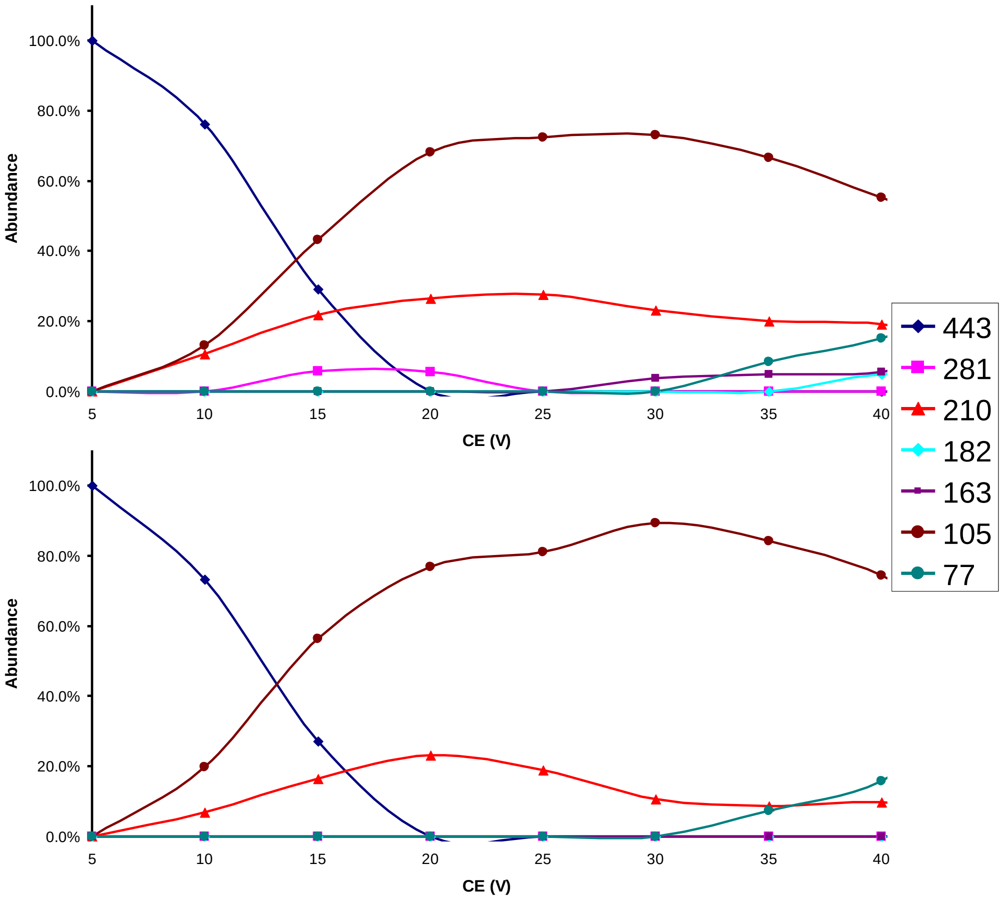

SF7: Breakdown curves for 7a-7b isomer pairs.

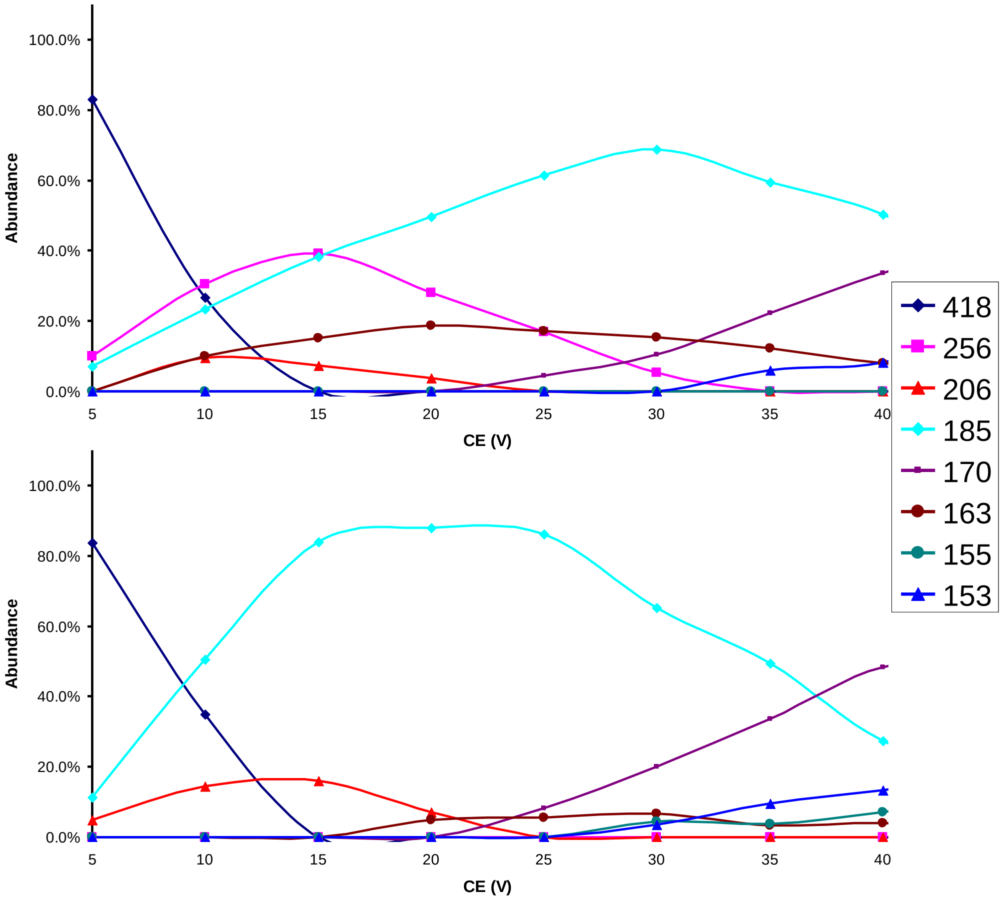

SF8: Breakdown curves for 8a-8b isomer pairs.

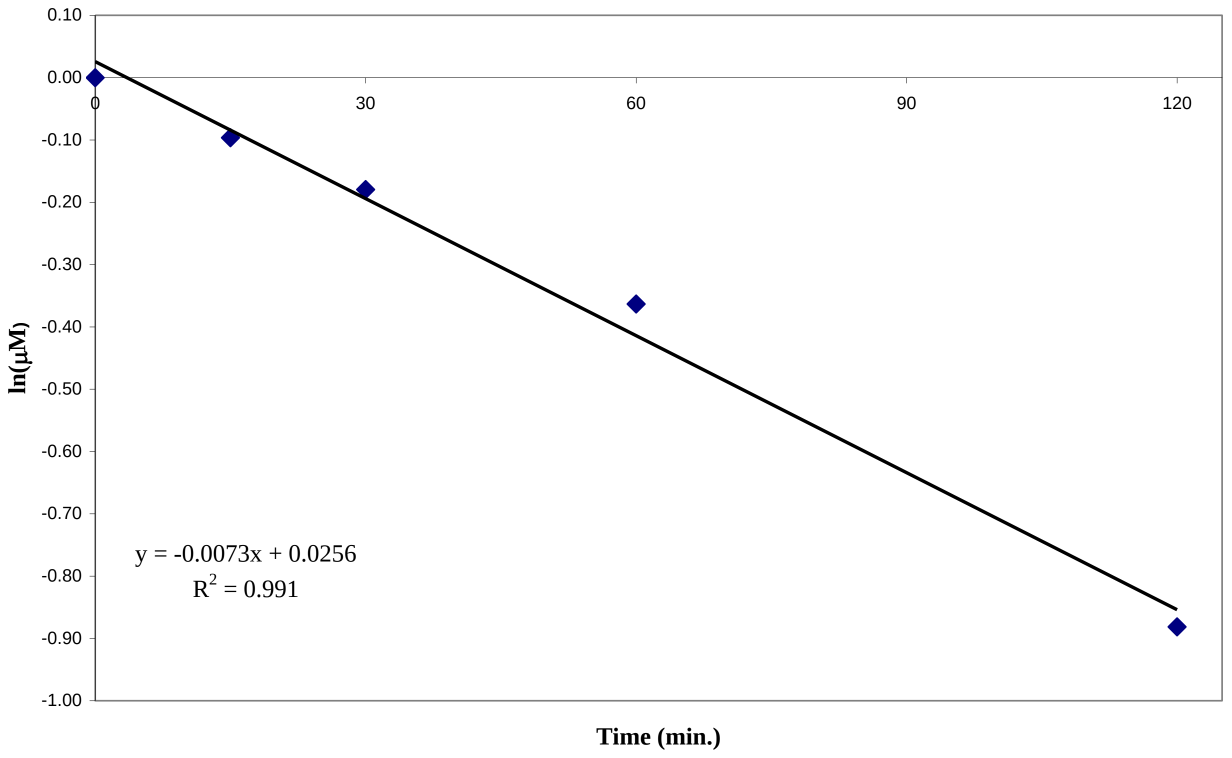

SF9: Degradation plot of KEE in human plasma samples.

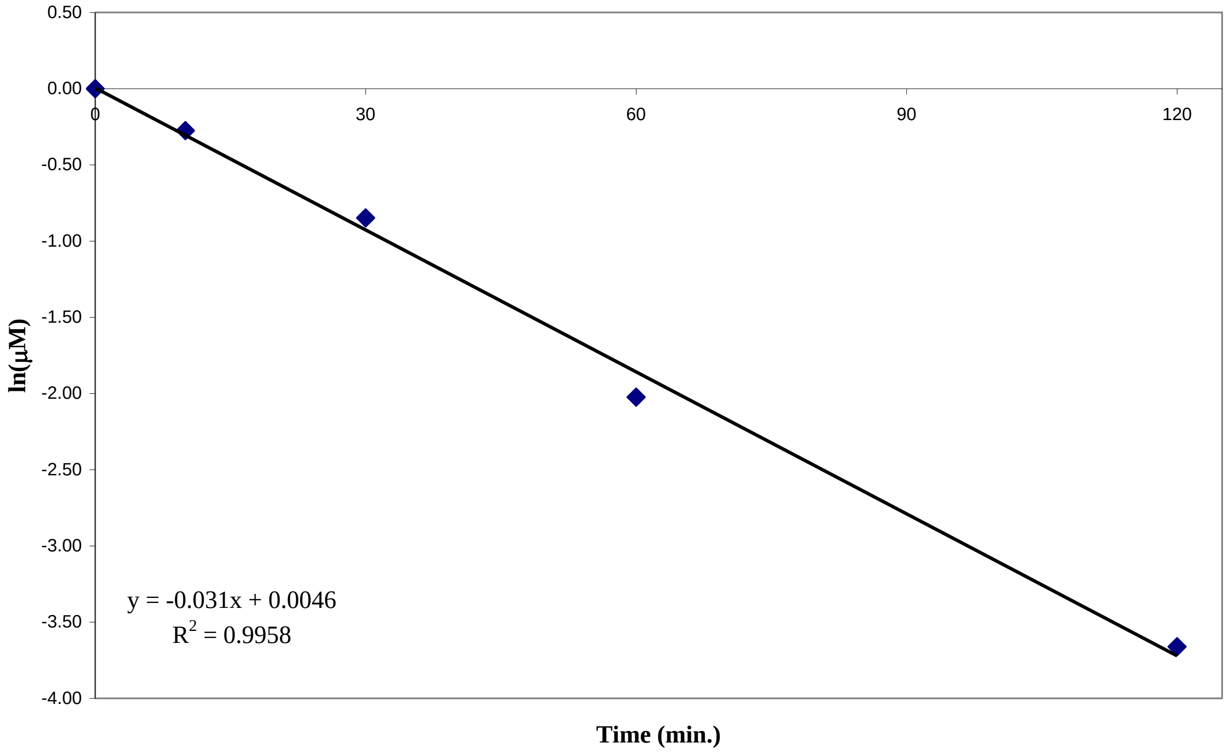

SF10 : degradation plot of Enalapril in rat plasma samples.

ST2: Half life of references and studied compounds.

	PBS<br>T 1/2 (min)	Human plasma<br>T 1/2 (min)	Rat plasma<br>T 1/2 (min)
KEE	n.d	95	n.d
Enalapril	n.d	n.d	22
1A	>240	>240	>240
1B	>240	>240	>240
2A	>240	>240	>240
2B	>240	>240	>240
3A	>240	>240	>240
3B	>240	>240	>240
4A	>240	>240	>240
4B	>240	>240	>240
5A	>240	>240	>240
5B	>240	>240	>240
6A	>240	>240	>240
6B	>240	>240	>240
7A	>240	>240	>240
7B	>240	>240	>240
8A	>240	>240	>240
8B	>240	>240	>240

n.d.: not determinated

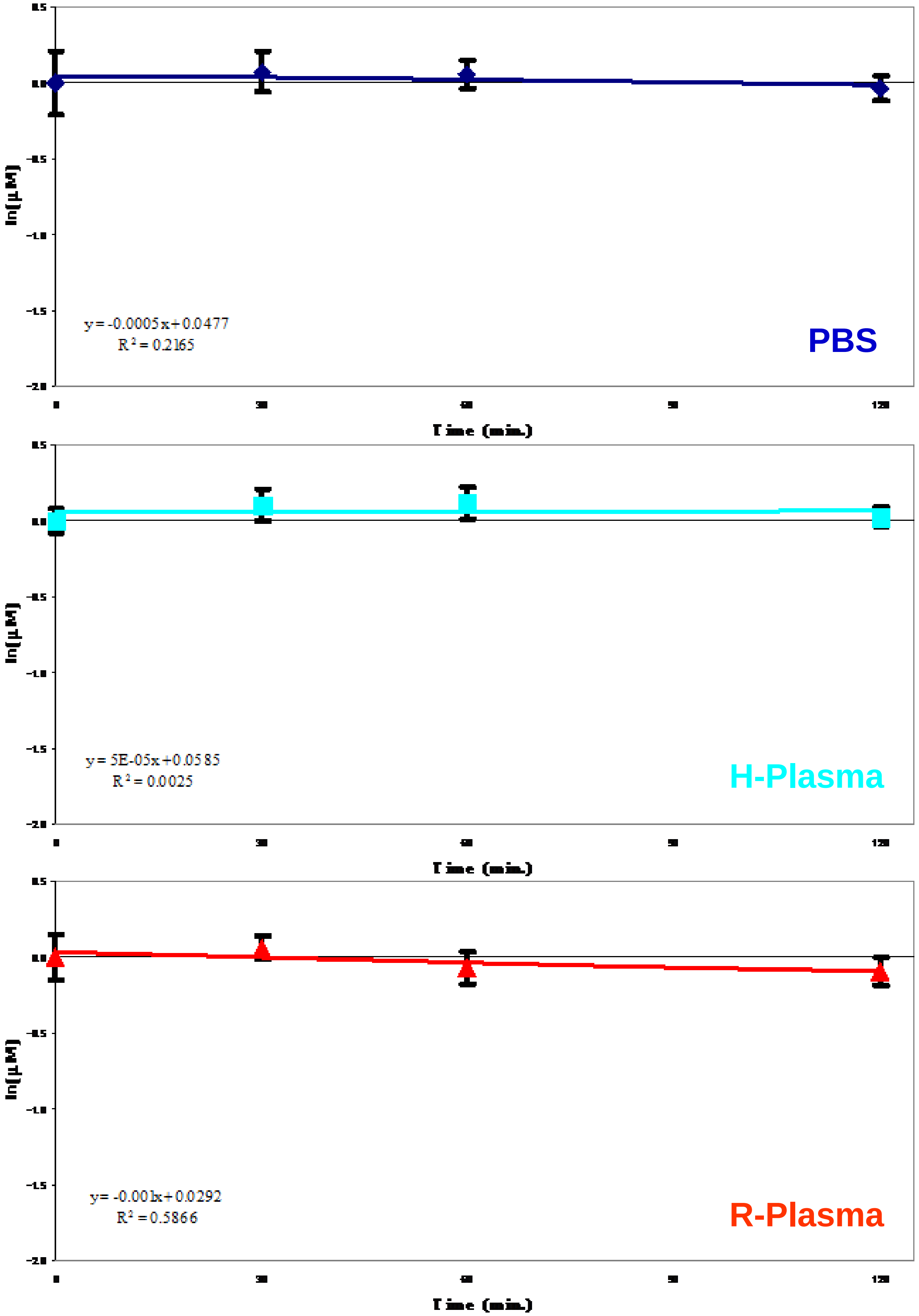

SF11: degradation plot of compound 1a in phosphate buffer solution (PBS), human (H-Plasma) and rat (R-Plasma) plasma samples.

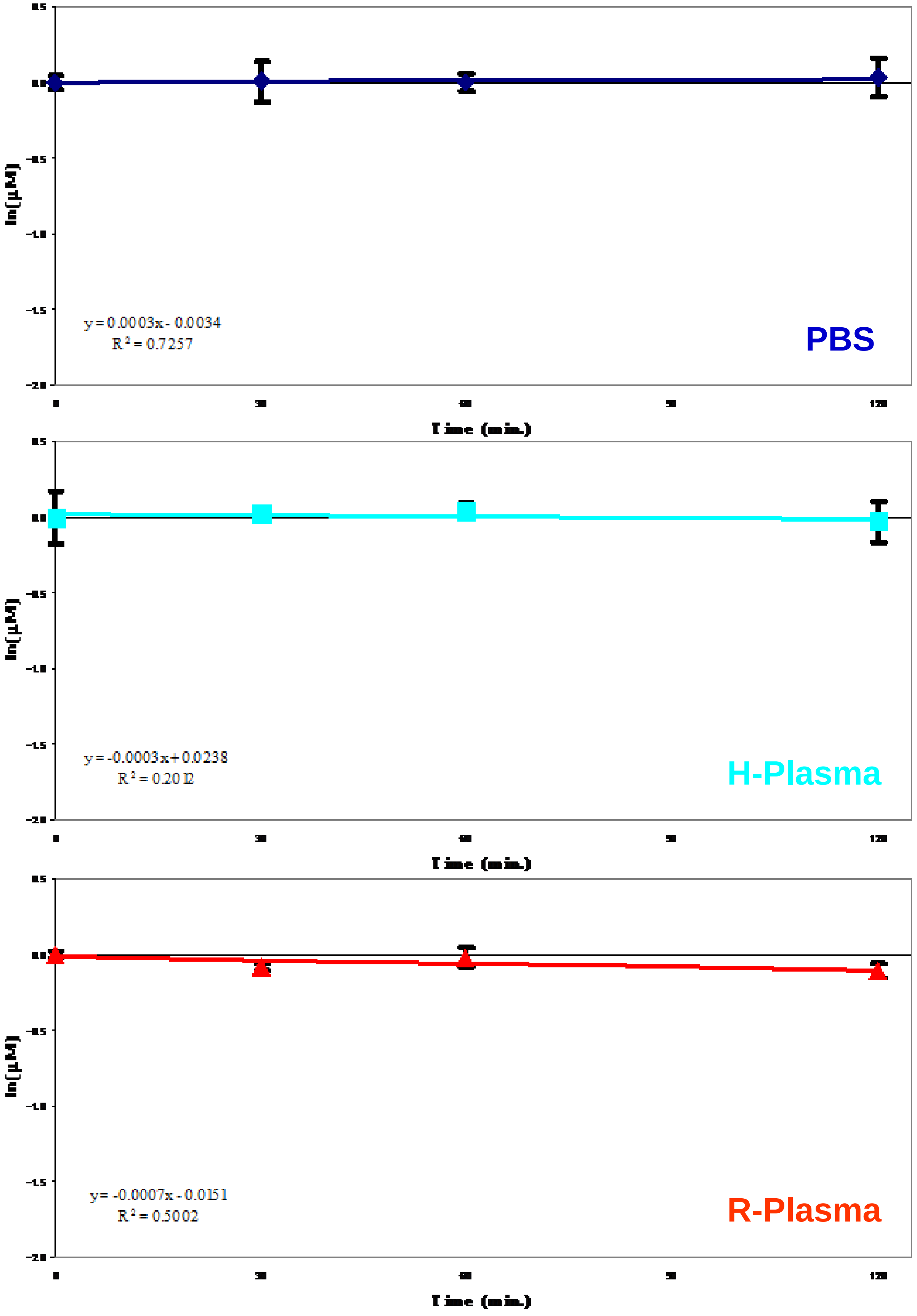

SF12: degradation plot of compound 1b in phosphate buffer solution (PBS), human (H-Plasma) and rat (R-Plasma) plasma samples.

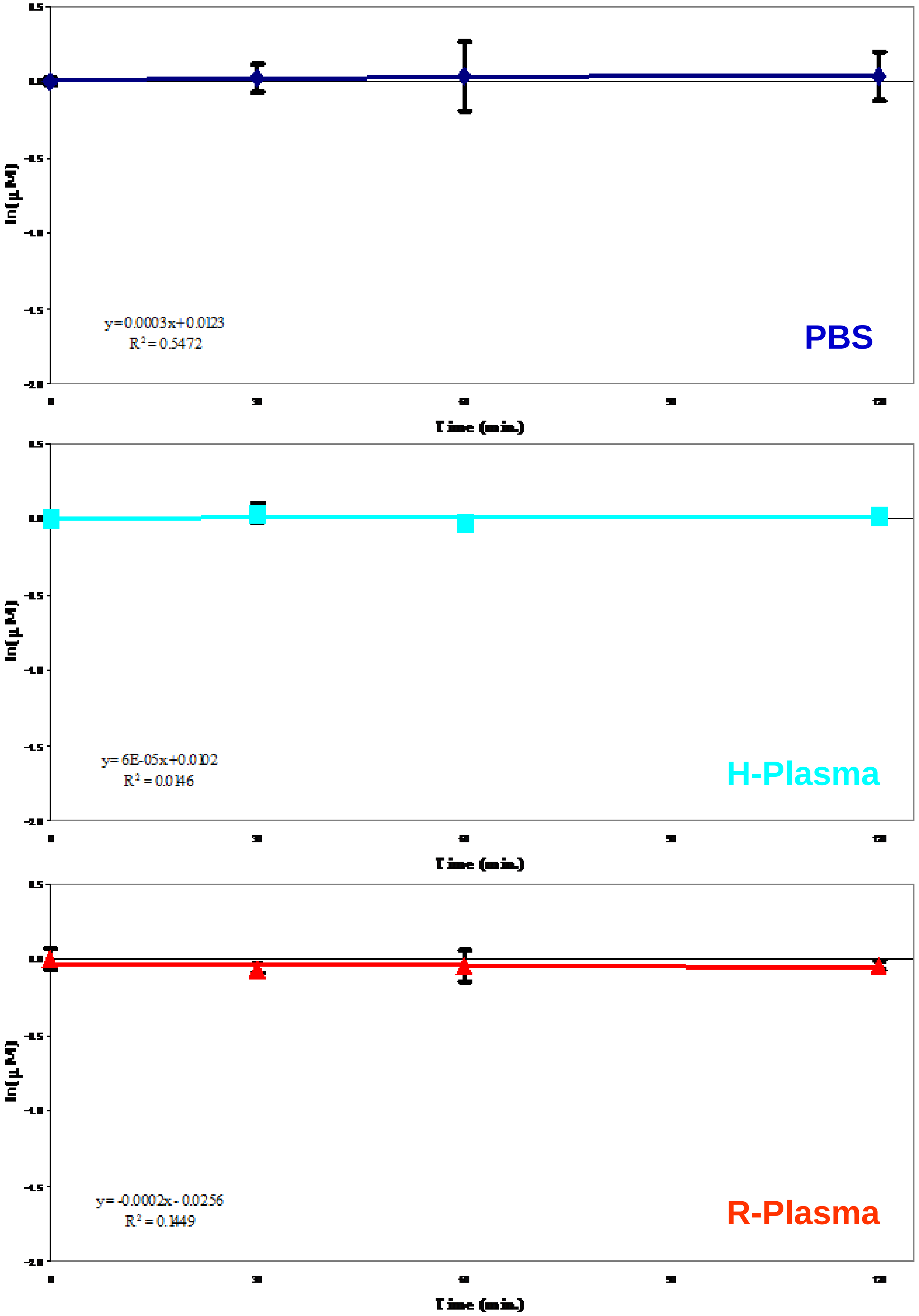

SF13: degradation plot of compound 2a in phosphate buffer solution (PBS), human (H-Plasma) and rat (R-Plasma) plasma samples.

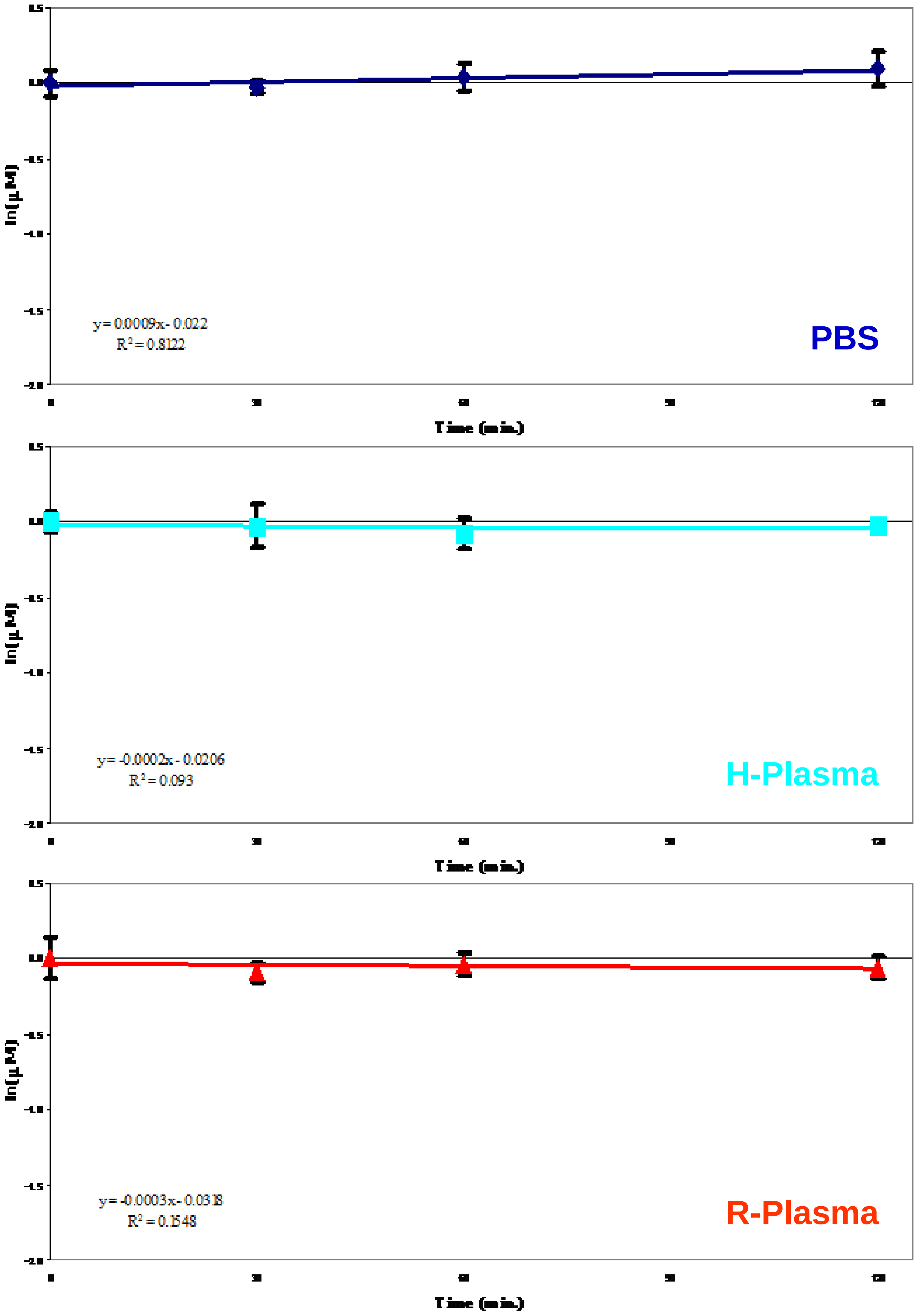

SF14: degradation plot of compound 2b in phosphate buffer solution (PBS), human (H-Plasma) and rat (R-Plasma) plasma samples.

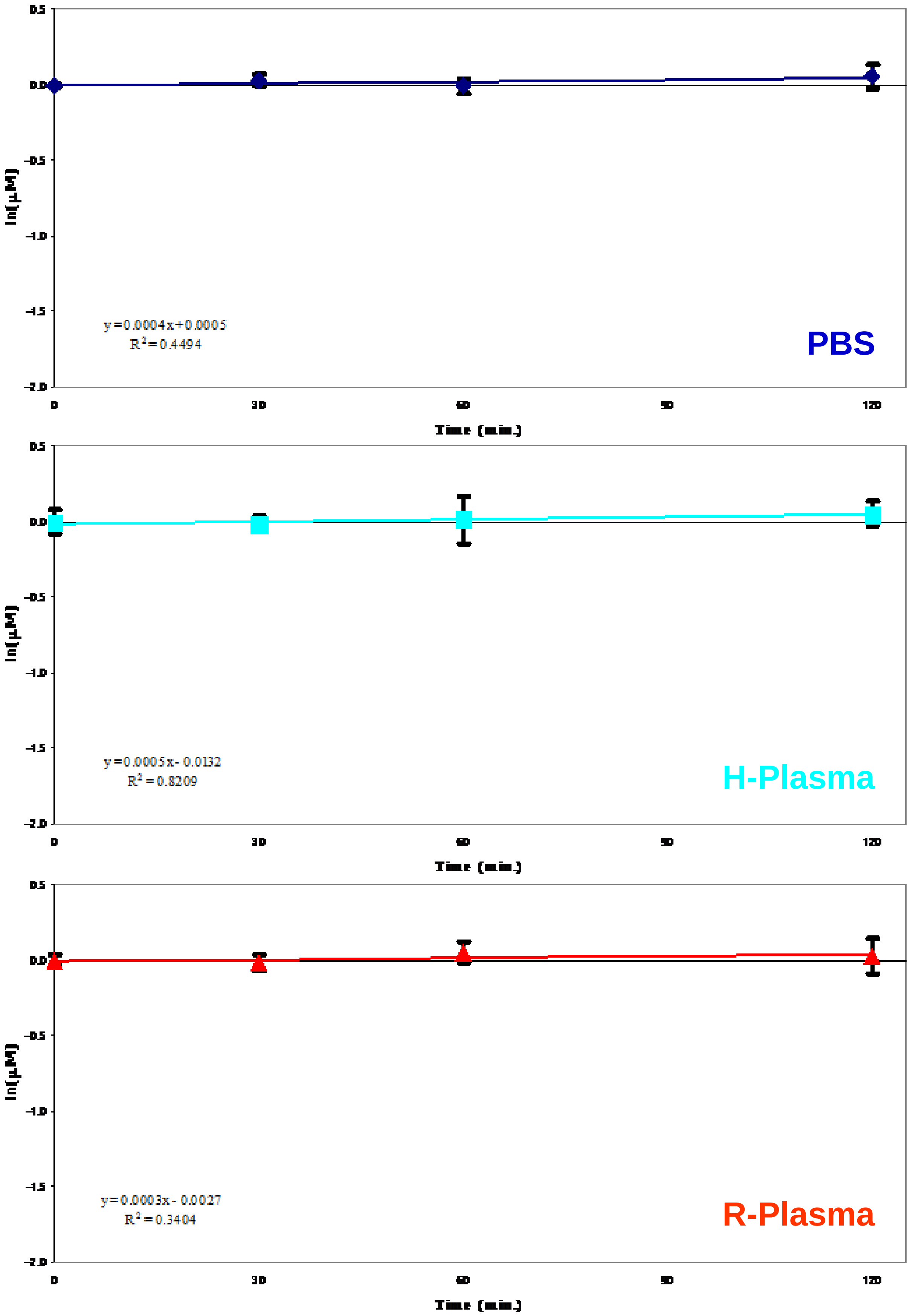

SF15: degradation plot of compound 3a in phosphate buffer solution (PBS), human (H-Plasma) and rat (R-Plasma) plasma samples.

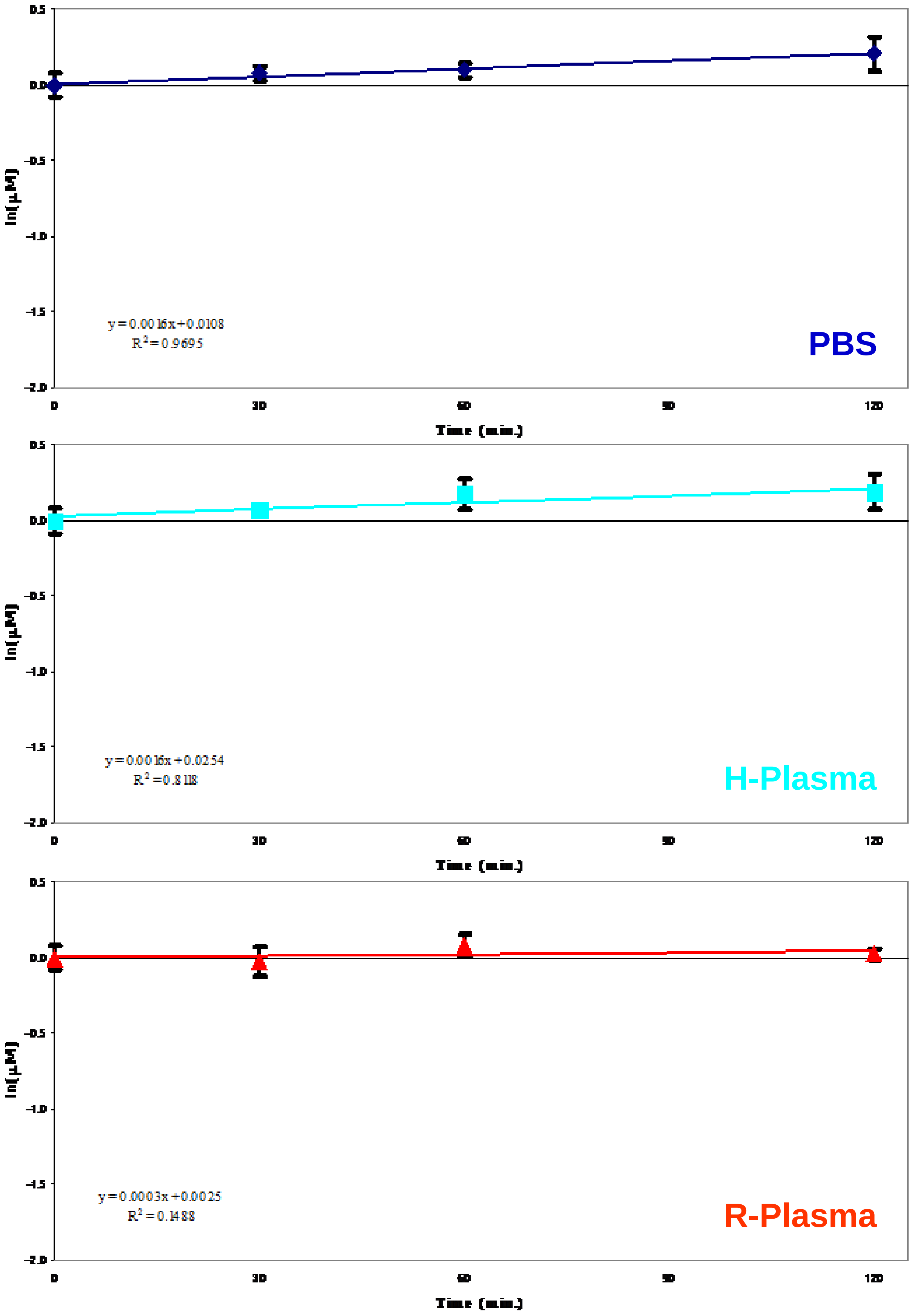

SF16: degradation plot of compound 3b in phosphate buffer solution (PBS), human (H-Plasma) and rat (R-Plasma) plasma samples.

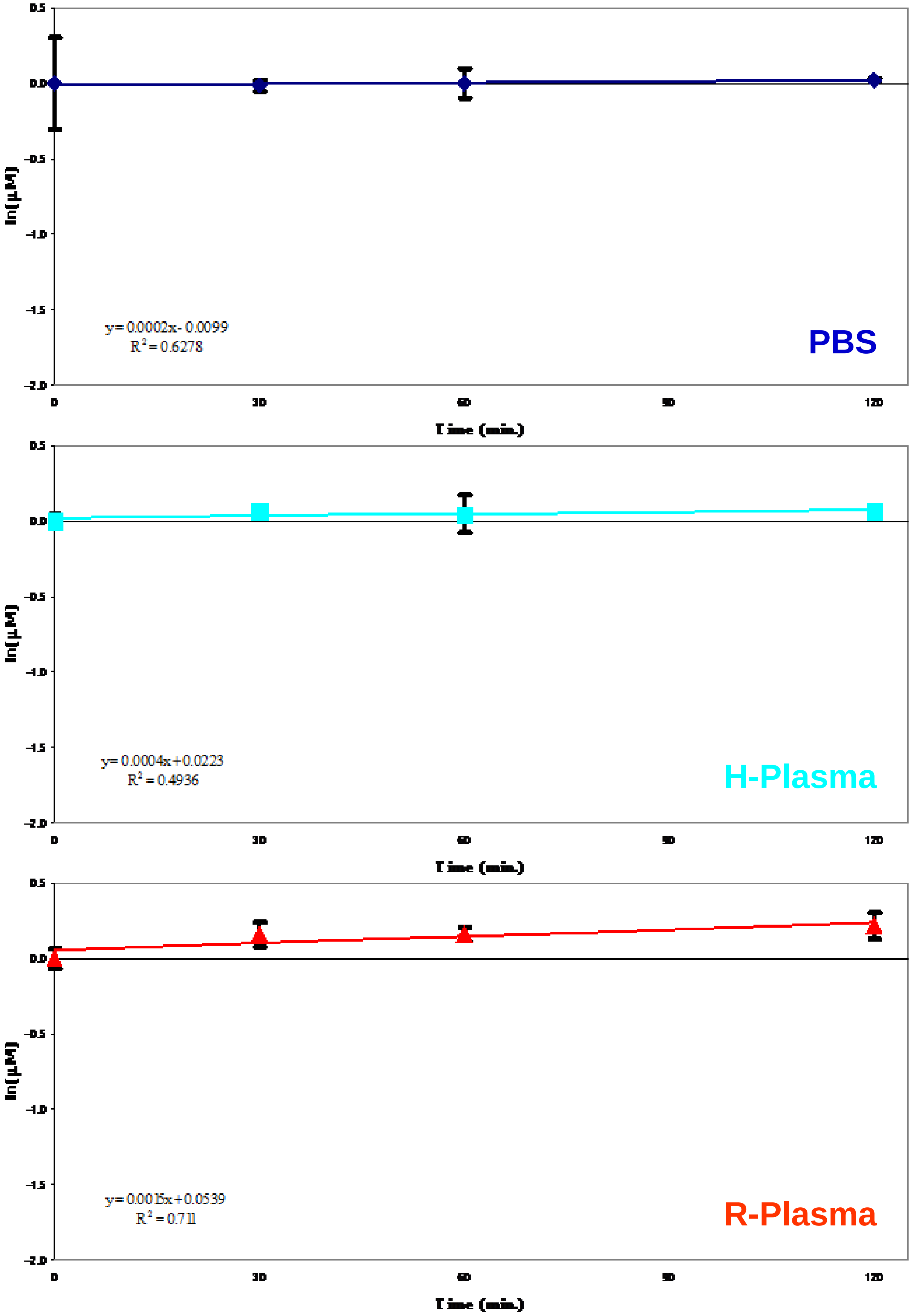

SF17: degradation plot of compound 4a in phosphate buffer solution (PBS), human (H-Plasma) and rat (R-Plasma) plasma samples.

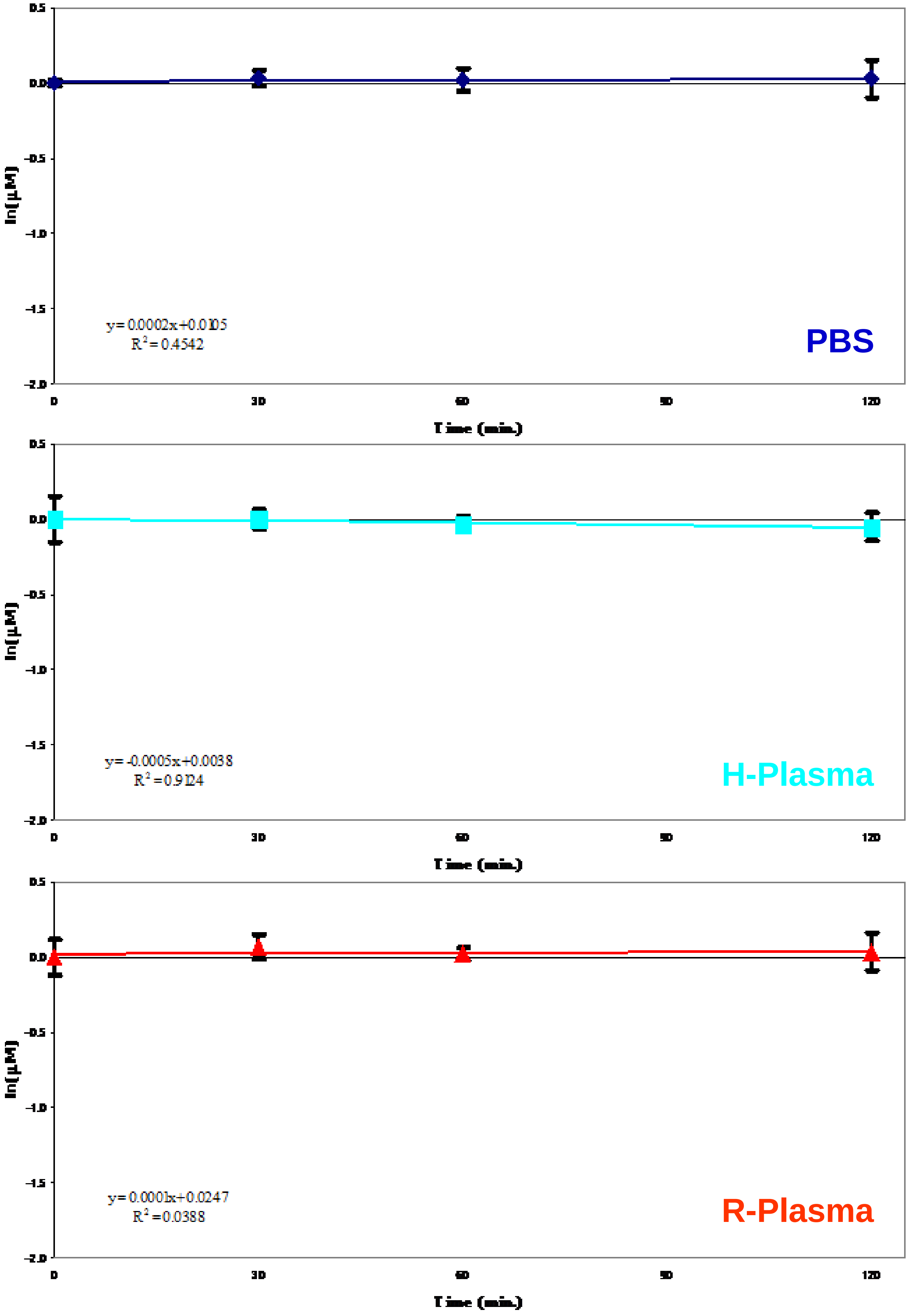

SF18: degradation plot of compound 4b in phosphate buffer solution (PBS), human (H-Plasma) and rat (R-Plasma) plasma samples.

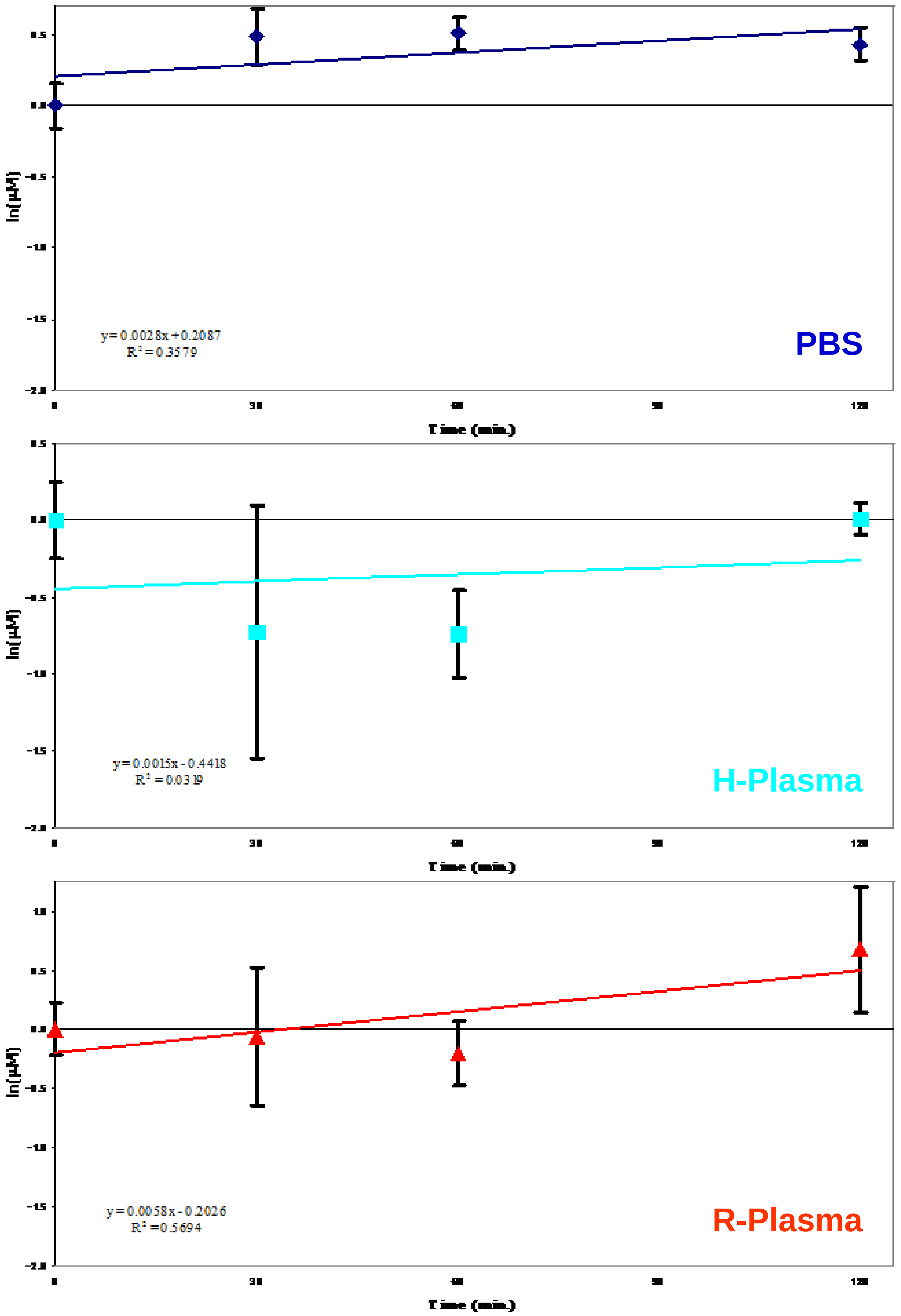

SF19: degradation plot of compound 5a in phosphate buffer solution (PBS), human (H-Plasma) and rat (R-Plasma) plasma samples.

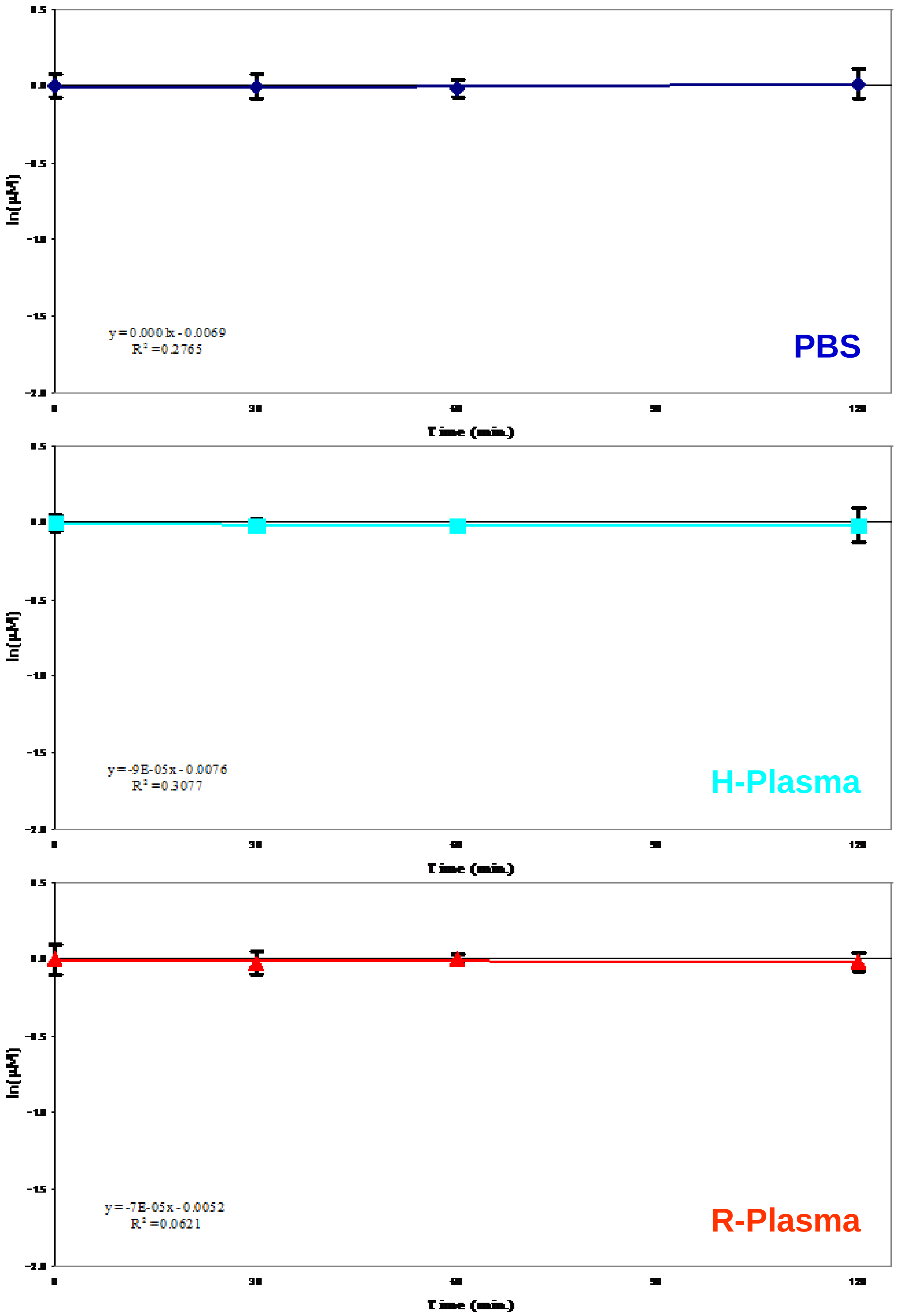

SF20: degradation plot of compound 5b in phosphate buffer solution (PBS), human (H-Plasma) and rat (R-Plasma) plasma samples.

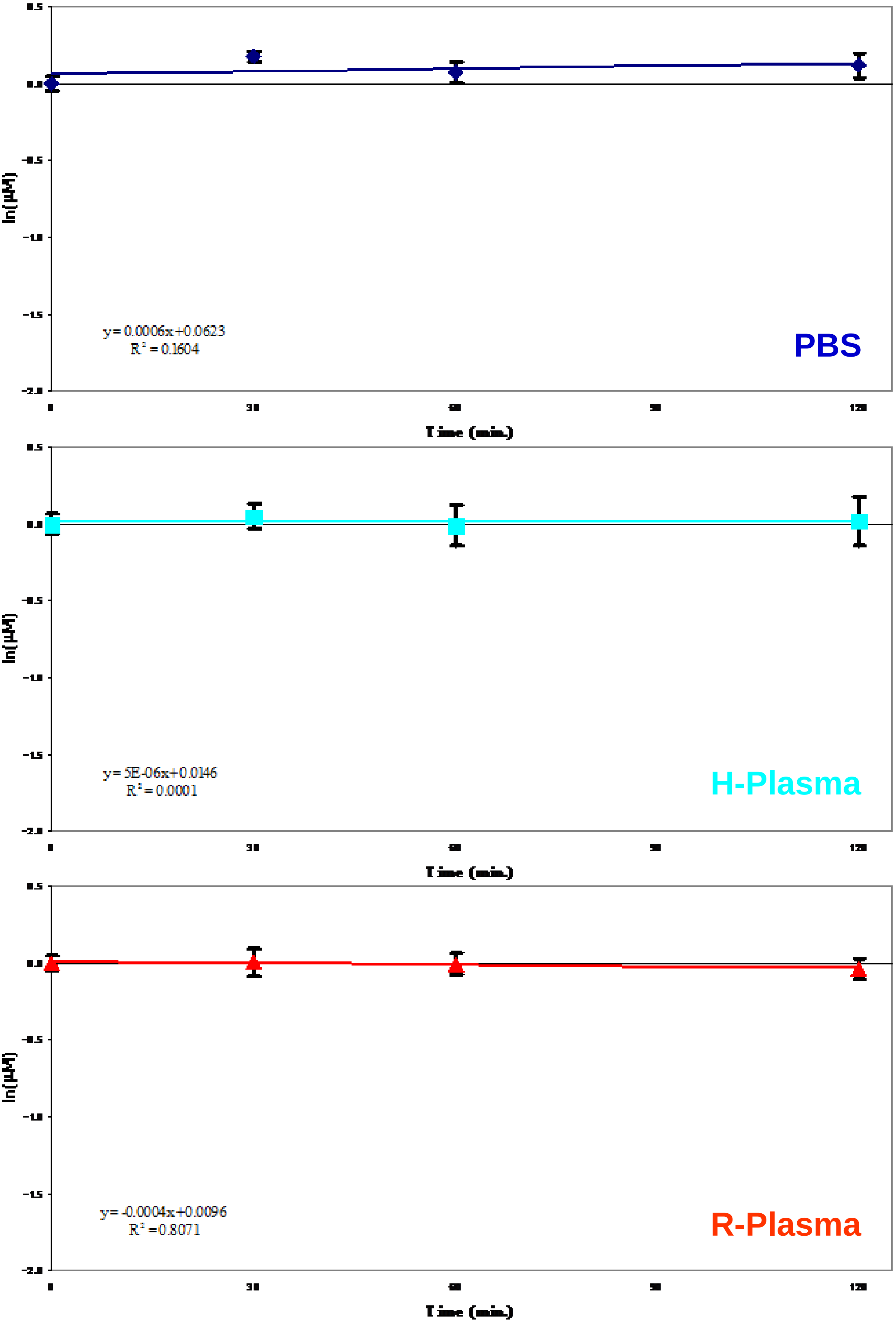

SF21: degradation plot of compound 6a in phosphate buffer solution (PBS), human (H-Plasma) and rat (R-Plasma) plasma samples.

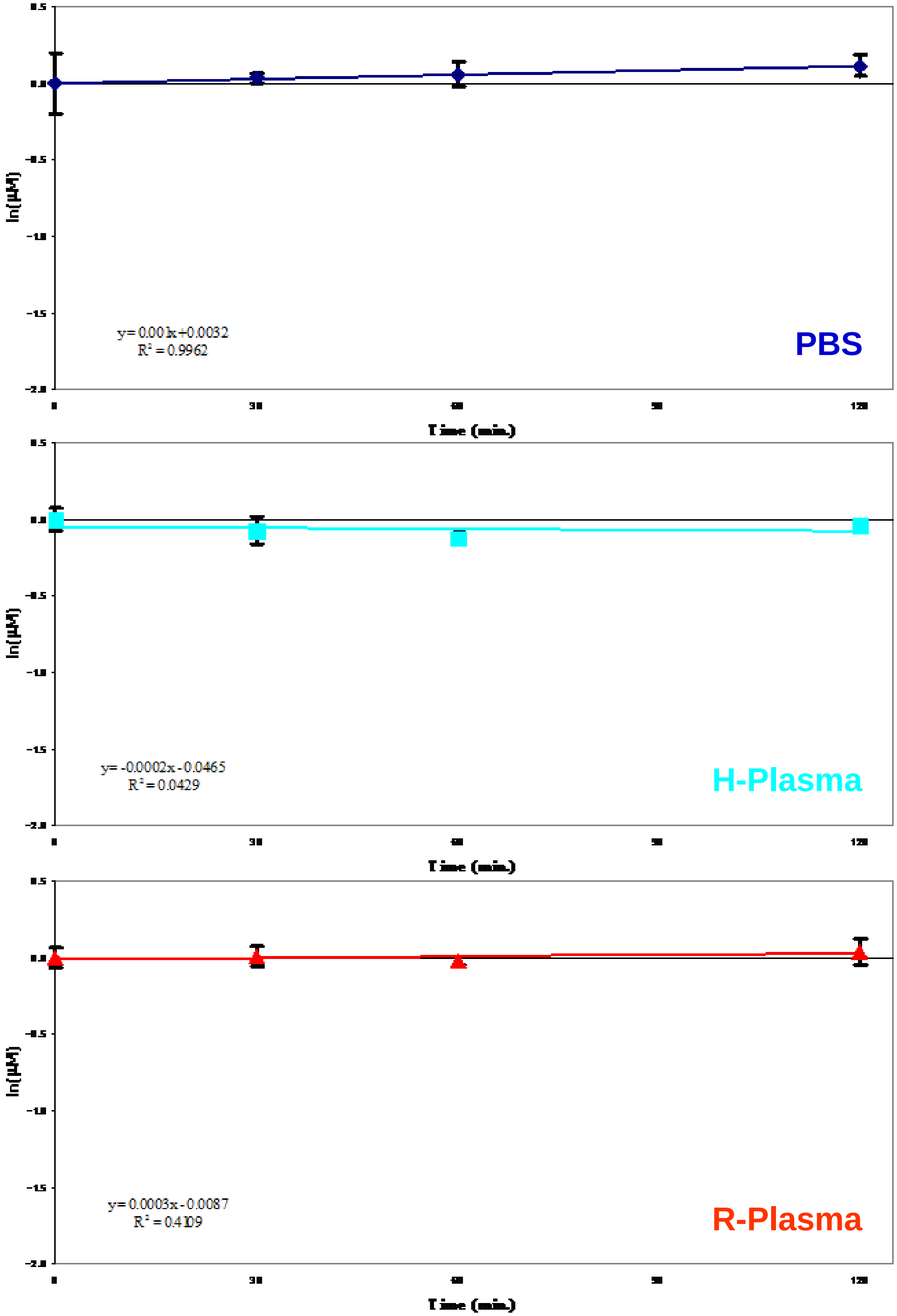

SF22: degradation plot of compound 6b in phosphate buffer solution (PBS), human (H-Plasma) and rat (R-Plasma) plasma samples.

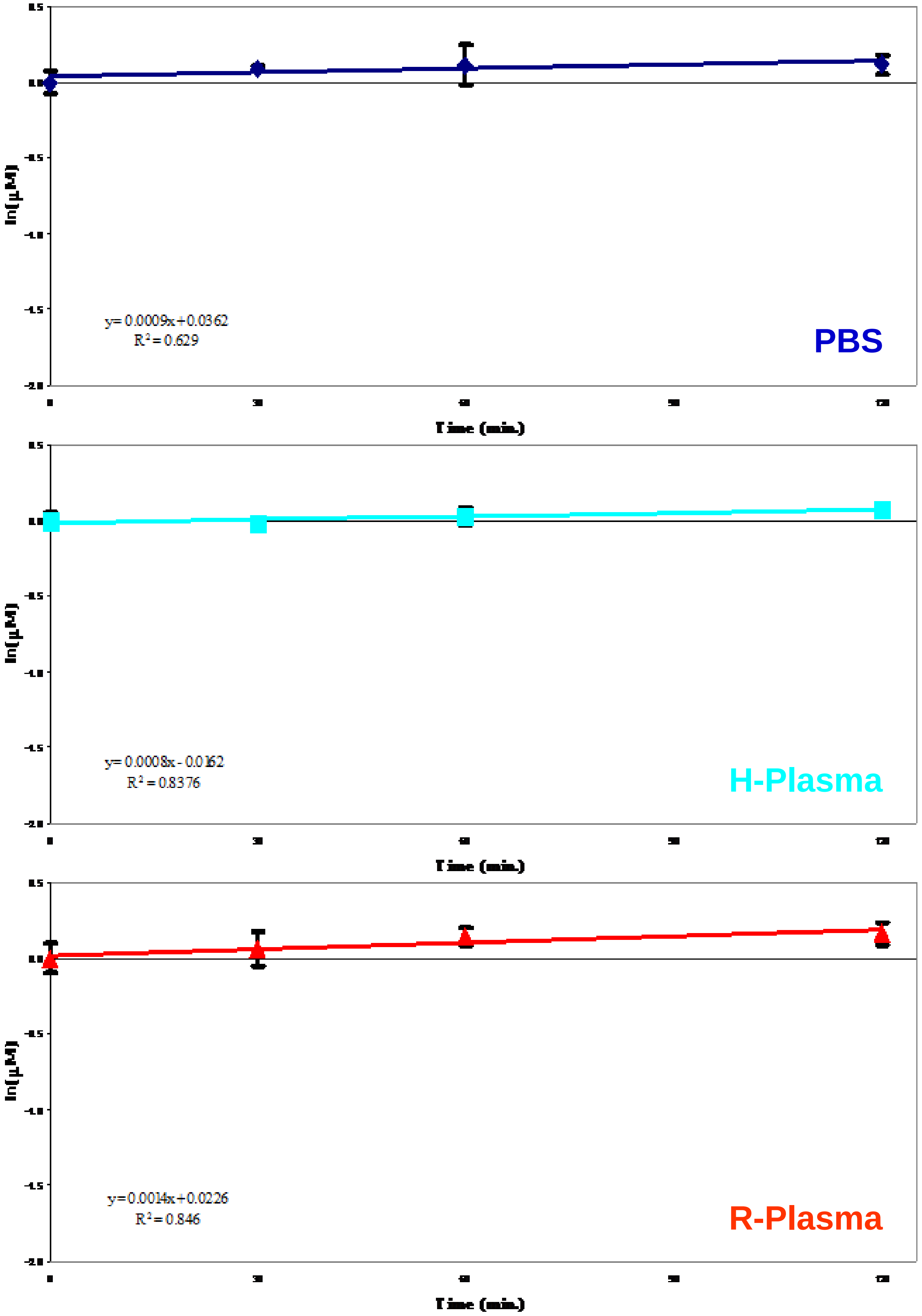

SF23: degradation plot of compound 7a in phosphate buffer solution (PBS), human (H-Plasma) and rat (R-Plasma) plasma samples.

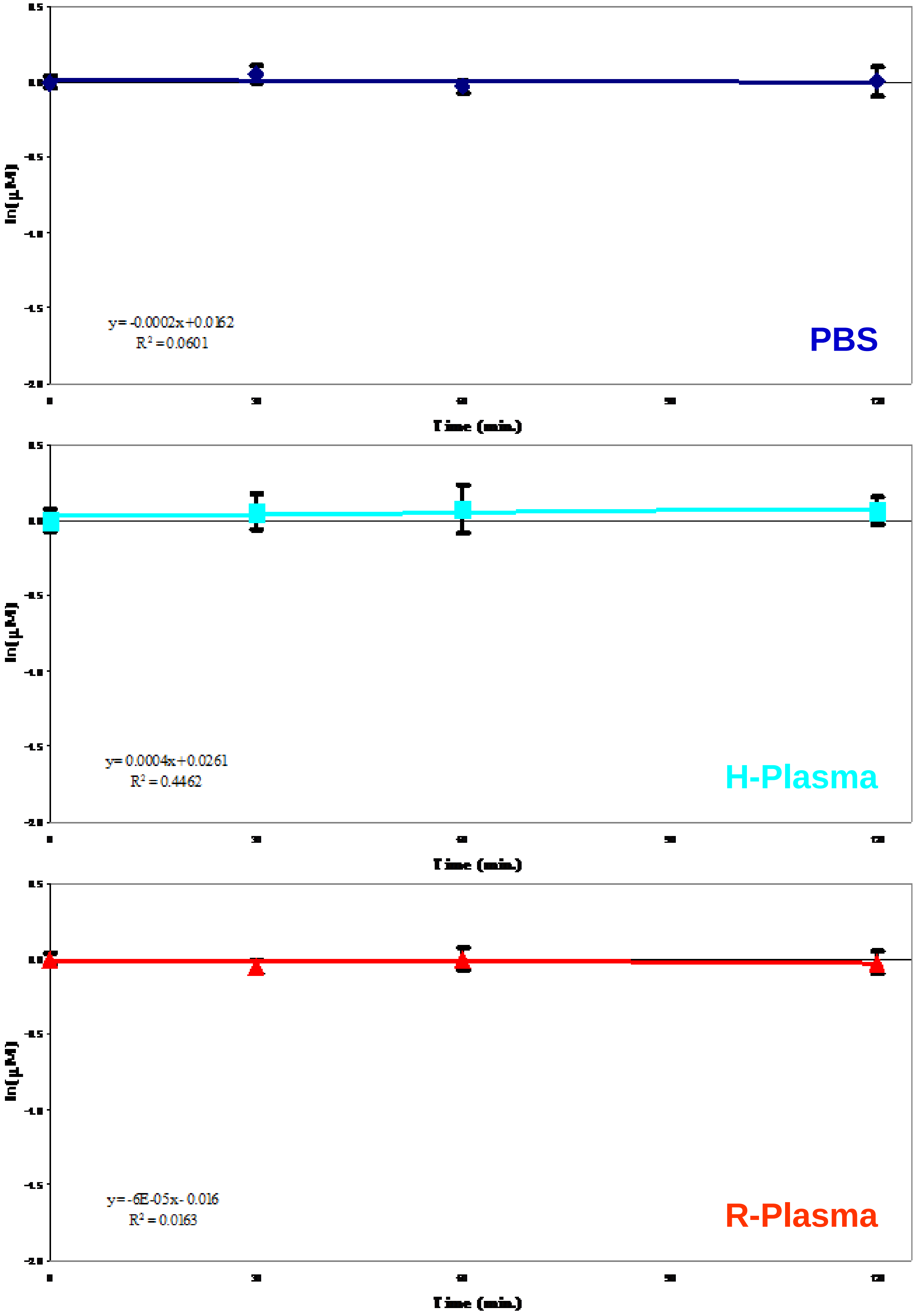

SF24: degradation plot of compound 7b in phosphate buffer solution (PBS), human (H-Plasma) and rat (R-Plasma) plasma samples.

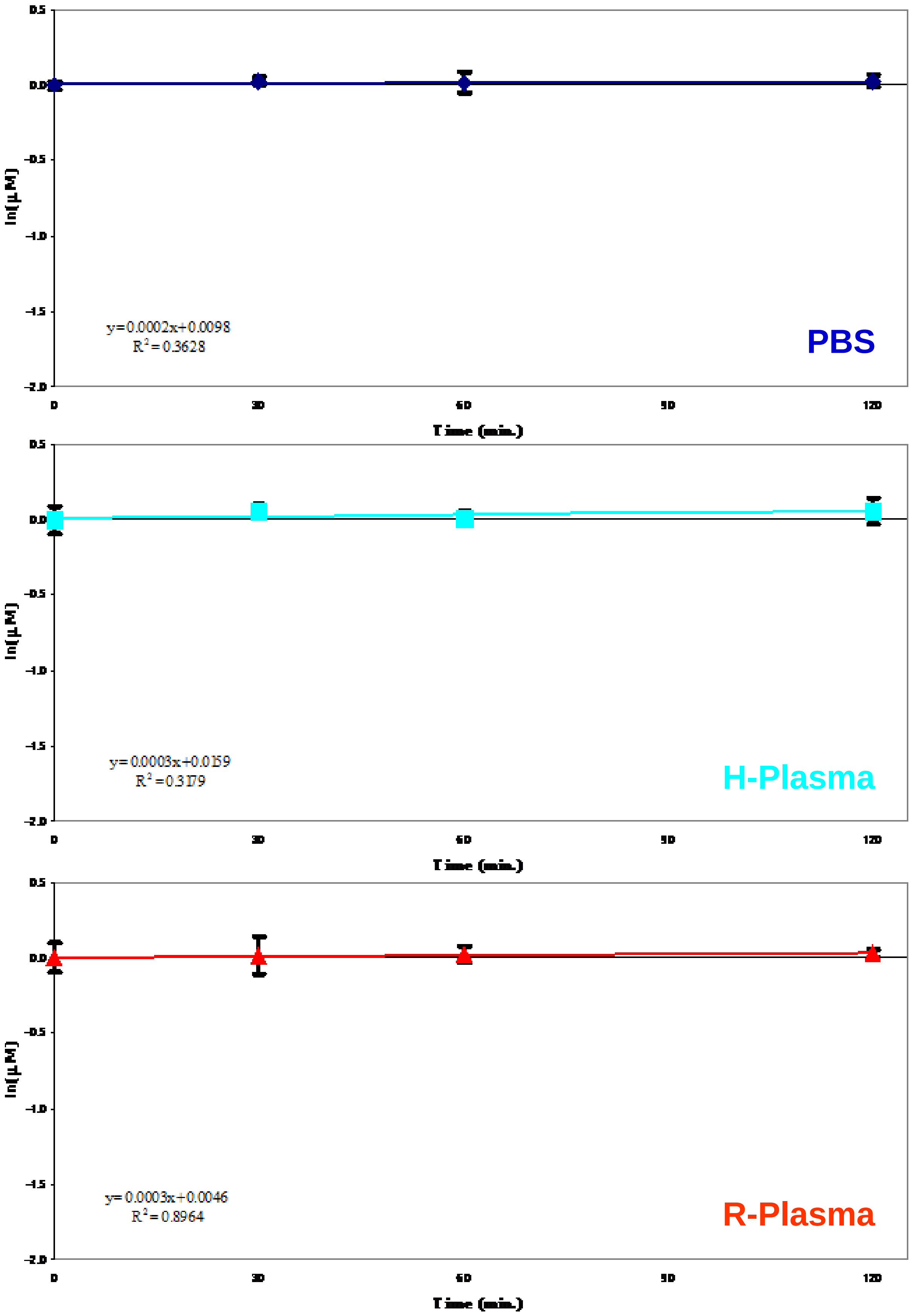

SF25: degradation plot of compound 8a in phosphate buffer solution (PBS), human (H-Plasma) and rat (R-Plasma) plasma samples.

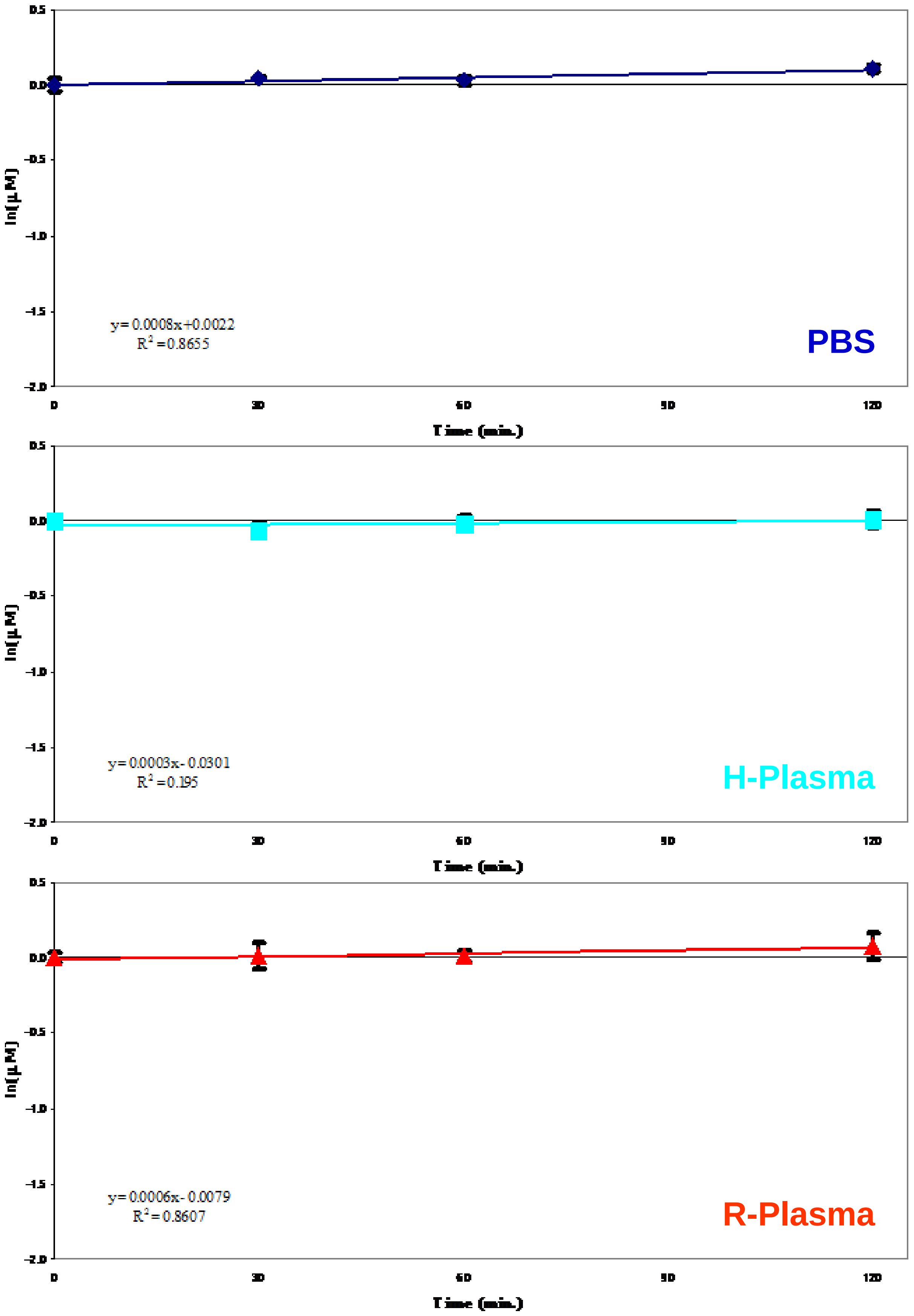

SF26: degradation plot of compound 8b in phosphate buffer solution (PBS), human (H-Plasma) and rat (R-Plasma) plasma samples.
